# A new major QTL for flag leaf thickness in barley (*Hordeum vulgare* L.)

**DOI:** 10.1186/s12870-022-03694-7

**Published:** 2022-06-24

**Authors:** Yanan Niu, Tianxiao Chen, Zhi Zheng, Chenchen Zhao, Chunji Liu, Jizeng Jia, Meixue Zhou

**Affiliations:** 1 Tasmanian Institute of Agriculture, University of Tasmania, Private Bag 1375, 7250 Prospect, TAS Australia; 2 CSIRO Agriculture and Food, 4067 St Lucia, QLD Australia; 3 National Key Facility for Crop Gene Resources and Genetic Improvement, Institute of Crop Sciences, Chinese Academy of Agricultural Sciences, 100081 Beijing, China; 4 College of Agronomy, Shanxi Agricultural University, 030801 Taigu, China

**Keywords:** Wild barley, QTL mapping, Flag leaf, Yield, Genetic improvement

## Abstract

**Background:**

Carbohydrate accumulation of photosynthetic organs, mainly leaves, are the primary sources of grain yield in cereals. The flag leaf plays a vital role in seed development, which is probably the most neglected morphological characteristic during traditional selection processes.

**Results:**

In this experiment, four flag leaf morphological traits and seven yield-related traits were investigated in a DH population derived from a cross between a wild barley and an Australian malting barley cultivar. Flag leaf thickness (FLT) showed significantly positive correlations with grain size. Four QTL, located on chromosomes 1H, 2H, 3H, and 5H, respectively, were identified for FLT. Among them, a major QTL was located on chromosome 3H with a LOD value of 18.4 and determined 32% of the phenotypic variation. This QTL showed close links but not pleiotropism to the previously reported semi-dwarf gene *sdw1* from the cultivated barley. This QTL was not reported before and the thick leaf allele from the wild barley could provide a useful source for improving grain yield through breeding.

**Conclusions:**

Our results also provided valuable evidence that source traits and sink traits in barley are tightly connected and suggest further improvement of barley yield potential with enhanced and balanced source and sink relationships by exploiting potentialities of the wild barley resources. Moreover, this study will provide a novel sight on understanding the evolution and development of leaf morphology in barley and improving barley production by rewilding for lost superior traits during plant evolution.

**Supplementary Information:**

The online version contains supplementary material available at 10.1186/s12870-022-03694-7.

## Background

Barley (*Hordeum vulgare* L.) is one of the oldest cultivated cereals and is widely distributed in all agricultural regions [1]. Barley is also an important cereal which fulfils the increasing demand of raw materials for livestock food production, fermentable material for beer and certain distilled beverages, and as a component of various health foods [2–4]. Individual barley grain yield is the cumulative result of both source and sink strength for photoassimilates and nutrients over the course of seed development, where the source potentially reflects photosynthetic capacity, and the sink shows the potential capacity to accumulate photosynthate [5]. Strengthening source and sink and modifying sink-source relations could optimise crop yield. This is evidenced by the changes in source/sink of several crops during their domestication and transformation into modern cultivars, including rice, wheat, and barley [6–8].

Sources and sinks for both carbon and nitrogen are key components of plant productivity and yield, source–sink interactions are regulated by feedback, feedforward and crosstalk mechanisms [9, 10]. As the most important resource tissue, plant leaves are responsible for light interception, photosynthesis, and assimilate storage thus play vital roles in crop yield [11], determining over 50% carbohydrate accumulation in grains [12–14]. Leaf shapes arise within a developmental context that constrains both their evolution and environmental plasticity [15]. A leaf represents an investment on the part of a plant with the return being reflected by net dry-mass gain per unit leaf area. A leaf return will stop when photosynthesis no longer exceeds the costs of leaf respiration and root and stem activity to support the leaf’s photosynthesis [16].

A leaf’s physical strength depends on both its thickness and its tissue density. Among all the morphological traits of leaves, flag leaf thickness was reported to be positively correlated with the single-leaf net photosynthetic rate (Pn) in determining grain yield in rice [17, 18] as thicker leaves have higher chlorophyll (Chl) contents, which are the main pigments involved in light capture for photosynthesis [19]. Leaf thickness may also contribute to improved leaf angle and curvature [20]. Rice varieties with high yield potential and greater responsiveness to applied nitrogen (N) exhibited thick leaves along with short sturdy stems [21]. Positive correlations have also been reported between leaf thickness and panicle traits, such as panicle length, grain density, grain weight per panicle, and the number of spikelets per panicle in rice [22]. Therefore, leaf thickness could be considered as an important index in high-yielding cultivars breeding [22]. Plant leaf area, which is composed of leaf length and width, significantly influences plant growth, development, yield, and quality by affecting photosynthetic assimilates [23–27]. Flag leaf length and width in wheat are positively correlated with yield components, such as spike number per plant and tiller number per plant [28, 29].

Crop yield could be dissected into several components: number of ears per ha, grain number per ear and thousand-grain weight. So far, many genes or QTL related to grain yield and yield components have been reported in barley [30–37]. However, relatively fewer QTL were reported in barley for net photosynthetic rate and leaf morphological traits [38–41] compared with rice [42–46] and wheat [28, 29, 47–51]. The association between leaf morphological traits and yield potential has not been fully discovered in barley.

Crop domestication is one of the key approaches leading to currently cultivated crops [52]. The process of domestication, i.e. from wild barley to cultivated barley, has resulted in gene loss or changes in gene regulation/activity (i.e. via variations in the coding sequence) which imposes constraints on our ability to further improve cultivated varieties [52]. Breeders have been focusing on morphological characteristics for sink-related traits, such as grain size, with less efforts on source-related traits, in particular leaf thickness which is hard to select in the field. Thus the thicker leaf alleles could be easily lost or neglected during domestication and selection. Leaf shape could be seen as a functional response of plants to changes in the environment, understanding the potential adaptive value of leaf shape, and the genetic and molecular approaches to manipulate it will prove to be invaluable in breeding the next generation of crops and sustainably maintaining biodiversity and crop yield in future climates [15].

Our preliminary results showed that a wild barley accession, SYR01 (*Hordeum spontaneum*), showed much thicker flag leaf than the cultivated barley, Gairdner (*Hordeum vulgare* L.). Thus, the DH (doubled haploid) population from the cross between SYR01 and Gairdner was selected to identify QTL and linked molecular markers for flag leaf thickness and their potential links with other flag leaf traits, plant height and grain size. As it is too hard to select leaf thickness in the field, these markers can assist in selecting thicker leaf thus improve the source of cultivated barley.

## Result

### Phenotypic variations and correlations

**Fig. 1:**
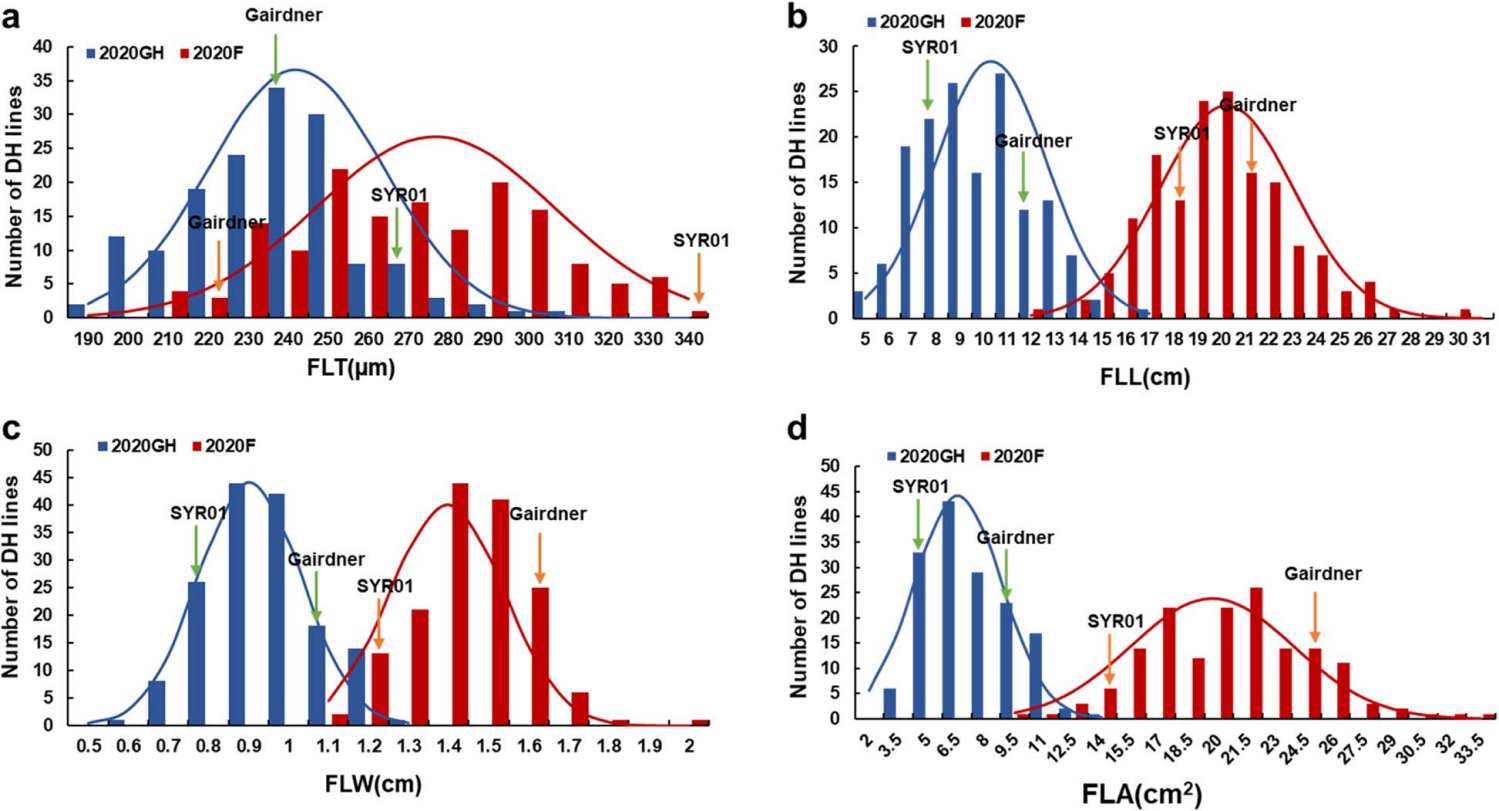
Frequency distribution of flag leaf traits in the DH population of SYR01 × Gairdner. Arrows indicate the phenotypes of SYR01 and Gairdner, respectively. FLT, flag leaf thickness; FLL, flag leaf length; FLW, flag leaf width; FLA, flag leaf area

Gairdner had a significantly longer, wider leaf and larger flag leaf area than SYR01 (Fig. 1). In contrast, SYR01 had a much thicker flag leaf than Gairdner (Fig. 1). All flag leaf traits showed large variations among DH lines across different years or trials, displaying continuous and near normal distribution (Fig. 1). Similar performances were also found in other traits (Supplementary Figures S1, S2). SYR01 showed taller plants, longer grains, while lower PL (panicle length), GWP (grain weight per panicle), TGW (thousand grain weight), GW (grain width), GT (grain thickness) and ASA (average seed area) than Gairdner (Supplementary Figures S1, S2). Field trials showed much thicker, longer and wider flag leaf than the glasshouse trials (Fig. 1) due to prolonged growth period in the field trials, suggesting a significant environmental effect on leaf development.

**Fig. 2:**
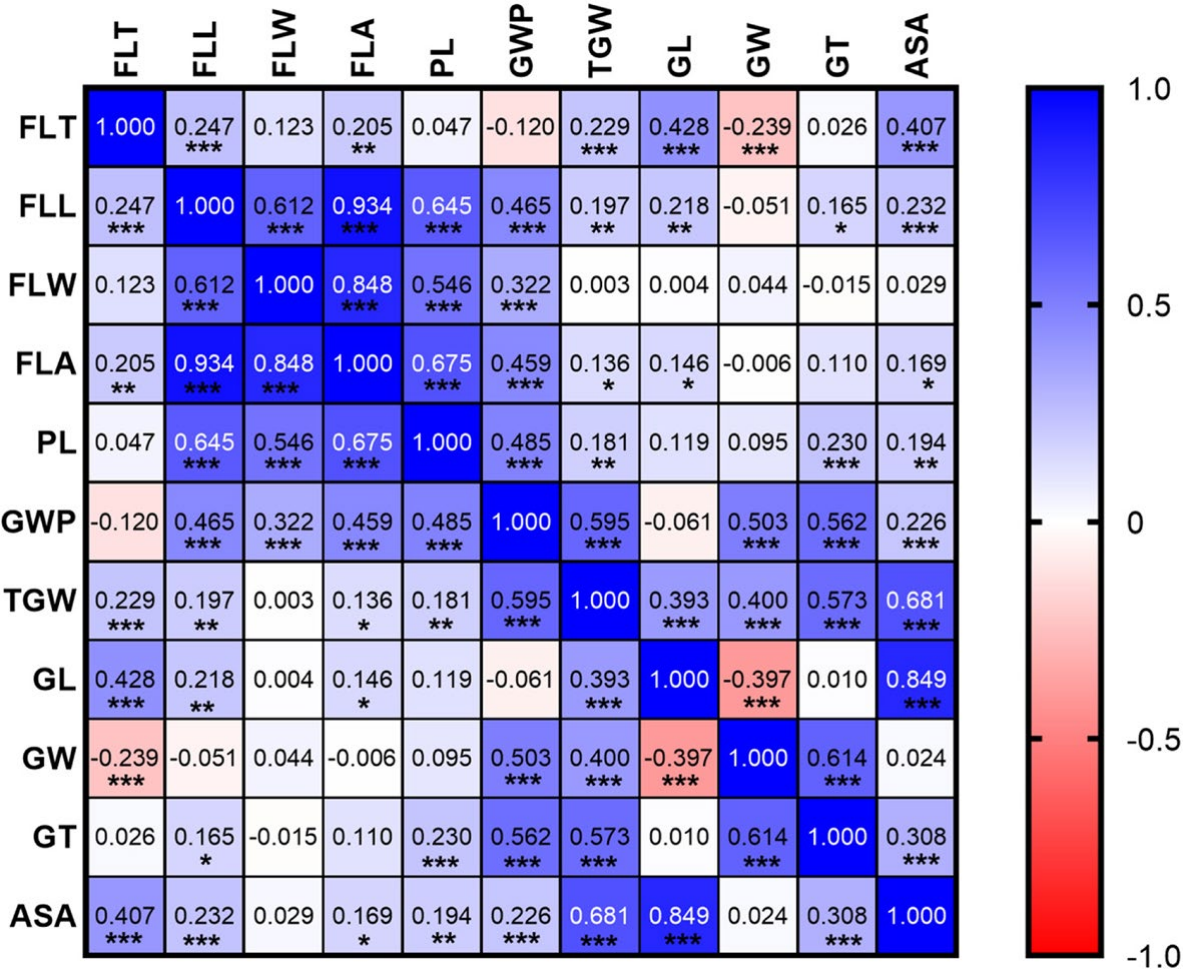
Correlations between flag leaf-related traits and yield-related traits. The number in the middle of the cell is the correlation coefficient; ‘*’, ‘**’ and ‘***’ refer to significant correlations (*P* < 0.05, *P* < 0.01 and *P* < 0.001). FLT, flag leaf thickness; FLL, flag leaf length; FLW, flag leaf width; FLA, flag leaf area; PL, panicle length; GWP, grain weight per panicle; TGW, thousand grain weight; GL, grain length; GW, grain width; GT, grain thickness, ASA, average seed area

From the average values of 2020 field and 2020 glasshouse trials, FLT (flag leaf thickness) showed weak but significant correlation with FLL (flag leaf length, *r* = 0.25, *P* < 0.01) and FLA (flag leaf area, *r* = 0.20, *P* < 0.05). FLT had a closer correlation with grain size than FLL and FLW (flag leaf width) while FLL and FLW were significantly correlated with PL and GWP (Fig. 2). All these leaf traits were highly inheritable with narrow-sense heritability ranging from 0.70 for FLT to 0.81 for FLL (Supplementary Table S1).

### QTL for flag leaf traits

**Fig. 3:**
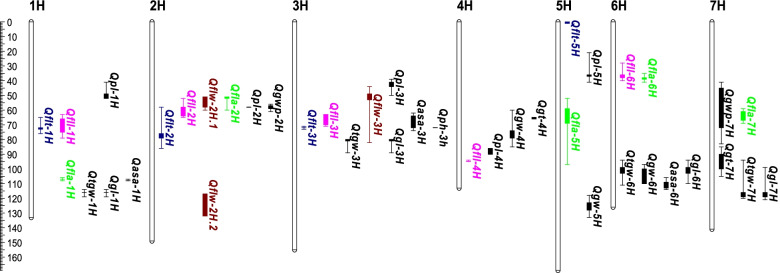
QTL for flag leaf traits in the DH population of SYR01 × Gairdner

QTL identified for flag leaf-related traits were widely distributed on all barley chromosomes (Table 1; Fig. 3). One major QTL for FLT was identified on chromosome 3H (*Qflt-3H*) with a LOD value of 18.4, determining more than 30% of the phenotypic variation. Three minor QTL (*Qflt-1H, Qflt-2H, Qflt-5H*) for FLT were located on chromosomes 1H, 2H, and 5H, respectively, determining 5.8 to 7.6% of the phenotypic variation. The wild barley contributed thicker leaf alleles for all the QTL. Five QTL (*Qfll-1H, Qfll-2H, Qfll-3H, Qfll-4H, Qfll-6H*) were identified for FLL, determining 5.8 to 17.9% of the phenotypic variation. *Qfll-2H* was located at a similar position to a minor QTL for FLT (*Qflt-2H*). Two QTL on chromosome 2H (*Qflw-2H.1, Qflw-2H.2*) were identified for FLW, determining 15.7 and 10.3% of the phenotypic variation, respectively. FLA was calculated from FLL and FLW, and the major QTL for FLA was located at the same positions of these QTL for FLL and/or FLW. These four QTL determined more than 50% of the phenotypic variation. At the genetic map position of 50–80 cM on chromosome 2H, QTL for all the flag leaf traits, FLT (*Qflt-2H*), FLL (*Qfll-2H*), FLW (*Qflw-2H.1*), and FLA (*Qfla-2H*), were identified (Table 1). This region is around the centromere. *Qflt-3H*, *Qfll-4H*, and *Qfla-2H* were identified in both field trials and glasshouse trials.

**Table 1:** QTL for flag leaf related traits and yield related traits

Traits^a^	QTL	Chr.	Position (cM)	Nearest marker	2-LOD interval (cM)	LOD	*R*^*2*^ (%)^b^	Additive effect^c^
FLT	*Qflt-1H*	1H	72.6	11118295D1	64.7–76.3	5.28	7.6	6.98
	*Qflt-2H*	2H	79.1	3811670D2	58.0-85.5	4.8	6.9	6.86
	*Qflt-3H*	3H	72.0	5,258,214 S	70.8–73.1	18.38	32.7	14.41
	*Qflt-5H*	5H	0.0	13142319D5	0-1.1	4.11	5.8	6.01
FLL	*Qfll-1H*	1H	70.5	3430654D1	63.0-78.6	4.06	5.8	0.60
	*Qfll-2H*	2H	65.0	4791044D2	51.7–65.0	11.44	17.9	-1.08
	*Qfll-3H*	3H	64.2	4007500D3	63.2–71.0	5.51	8.1	-0.74
	*Qfll-4H*	4H	94.8	4000059D4	94.2–94.6	10.37	16.4	-1.02
	*Qfll-6H*	6H	35.4	4415412D6	28.0–40.0	5.32	7.8	0.68
FLW	*Qflw-2H.1*	2H	51.7	5254141D2	51.2–60.3	5.70	15.7	-0.05
	*Qflw-2 H.2*	2H	117.7	6436762D2	116.8–132.0	3.62	10.3	-0.04
FLA	*Qfla-1H*	1H	107.1	3987047D1	106.0-108.0	7.68	13.3	1.01
	*Qfla-2H*	2H	51.7	5254141D2	50.7–60.3	13.66	25.9	-1.52
	*Qfla-6H*	6H	38.0	3930328D6	35.1–41.2	5.94	10.0	-0.95
	*Qfla-7H*	7H	64.0	4187271D7	59.0–69.0	3.62	5.9	1.07
PL	*Qpl-1H*	1H	48.6	3433594D1	41.0–52.0	7.69	10.4	0.36
	*Qpl-2H*	2H	58.3	100000209D2	58.0-58.4	15.56	23.8	-0.56
	*Qpl-3H*	3H	43.6	4188491D3	39.0–50.0	4.49	5.7	0.32
	*Qpl-4H*	4H	86.9	3271417D4	85.8–89.6	8.49	11.6	-0.38
	*Qpl-5H*	5H	36.6	3667033D5	21.0–41.0	3.47	4.4	0.24
TGW	*Qtgw-1H*	1H	115.8	7932258D1	113.8-118.7	5.79	11.4	1.65
	*Qtgw-3H*	3H	80.6	5256808D3	79.7–89.0	3.27	6.2	1.20
	*Qtgw-6H*	6H	101.0	3258371S6	94.0-111.0	5.54	10.8	-1.60
	*Qtgw-7H*	7H	116.4	3272131D7	94.0-120.0	3.67	7.0	-1.30
GL	*Qgl-1H*	1H	115.8	7932258D1	113.8-118.7	5.79	11.4	1.65
	*Qgl-3H*	3H	80.6	5256808D3	79.7–89.0	3.27	6.2	1.20
	*Qgl-6H*	6H	101.0	3258731S6	94.1-109.6	5.54	10.8	-1.60
	*Qgl-7H*	7H	116.4	3272131D7	99.0-121.0	3.67	7.0	-1.3
GW	*Qgw-4H*	4H	77.8	5248953D	60.0–77.0	3.26	7.3	-0.03
	*Qgw-5H*	5H	126.0	3257892D5	118.0-133.0	3.55	8.0	-0.03
	*Qgw-6H*	6H	106.3	3255067S6	97.0-110.0	5.46	12.6	-0.04
GT	*Qgt-4H*	4H	65.2	3259719D4	64.5-65.63	6.91	16.7	-0.03
	*Qgt-7H*	7H	96.1	3259168D7	85.0-105.0	3.5	8.1	-0.02
ASA	*Qasa-1H*	1H	108.1	3267839D1	107.7-108.4	12.97	26.3	0.70
	*Qasa-3H*	3H	67.9	3258789S3	62.0–74.0	4.22	7.5	0.38
	*Qasa-6H*	6H	111.3	15322541D7	106.0-114.0	5.95	10.8	-0.45
PH	*Qph-3H*	3H	72.0	3255135S3	71.9–72.1	31.01	62.9	14.72

^a^For trait abbreviations, see Figs. 1 and 2; PH: plant height

^b^Percentage of the phenotypic variation explained by the QTL

^c^Additive effect: positive values mean SYR01 alleles increased phenotypic values while negative values of the additive effect mean SYR01 alleles decreased trait scores

### QTL for grain size, PL and PH

Five QTL for PL were identified on chromosomes 1H, 2H, 3H, 4H, and 5H, respectively, explaining a total of 50% of phenotypic variation (Table 1). *Qpl-1H* and *Qpl-2H* were located at similar positions to two QTL (*Qflt-1H, Qflt-2H*) for FLT. Two QTL for TGW, GL, and GT, and three QTL for GW, and ASA were identified respectively (Table 1). The interval of the major QTL for GL (*Qgl-1H*) overlapped with *Qtgw-1H*, locating at similar positions to *Qasa-1H* and *Qfla-1H*. The allele from the wild parent SYR01 increased TGW, GL and ASA. The major QTL for TGW, *Qtgw-6H*, was located at a similar position to QTL for GW and ASA with Gairdner allele contributing the higher values. *Qgl-3H* for GL was located at a similar position to *Qasa-3H* for ASA on 3H. *Qgw-4H* for GW and *Qgt-4H* for GT are located at similar positions on 4H.

## Discussion

### Usefulness of FLT QTL

**Fig. 4:**
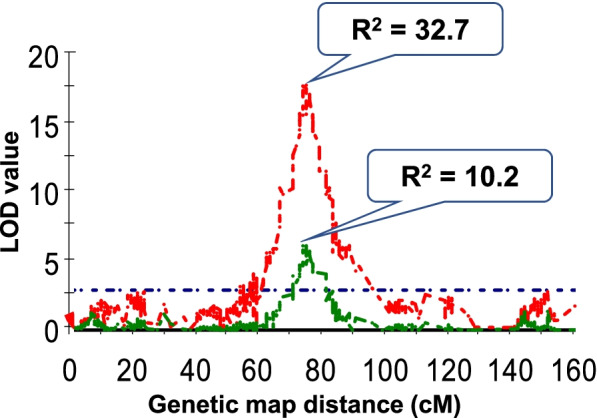
Changes of LOD and R^2^ of *Qflt-3H* before (red line) and after (green line) using plant height as a covariate

Morphological traits of flag leaf play important roles in determining crop grain yield and biomass, contributing a significant proportion of “the source” during grain filling stage. Many QTL have been reported for flag leaf length, width, and area [38, 40, 41]. These QTL are distributed on chromosomes 2H, 3H, 4H, 5H, 6H, and 7H. Our results also identified QTL for these traits on all the chromosomes (Fig. 3). According to the marker information in our study, *Qfll-2H* (55.7‒65.5 cM) is in similar regions to *qFLL2-2* and *qFLA2-2* for FLL and FLA, respectively, reported by Du et al. [38]. *Qflt-2H* (52.8‒73.7 cM), *Qflw-2H.1* (55.7‒67.1 cM) and *Qfla-2H* (56.7‒58.5 cM) for FLT, FLW and FLA, respectively, were also located in this region. *Qfla-5H* (51.8‒96.5 cM) for FLA in our study was likely the same locus to the QTL *qFW5.1* (44.1‒46.4 cM) for FLW reported earlier [41]. In a previous study, QTL “*D1Q1FLL2H*”, “*D1Q3FLL2H*” and “*D2Q4FLL2H*” for FLL [40] coincided with *Qflw-2H.2* (101.0‒139.2 cM) for FLW identified in our study. Zheng et al. [53] recently reported some QTL for FLT but the major QTL was different that identified in our study and the allele for increasing leaf thickness is from a cultivated barley. In this study, we have identified four QTL for FLT. The major one on 3H (631.9‒641.7 Mb) overlapped a semi-dwarf gene, *sdw1*(chr3H: 634,077,598–634,081,600) [54]. Our mapping results from this population also indicated a single major QTL, *Qph-3H* (*R*^*2*^ = 0.63), for plant height. This QTL was also located on 3H at a physical position of 633.98 Mb (72.0 cM), the same position of *Qflt-3H* for FLT. To further investigate the relationship between flag leaf thickness and plant height, we used plant height as a covariate to re-analyse QTL for FLT and the LOD value of *Qflt-3H* decreased from 16.91 to 6.33 and *R*^*2*^ reduced from 31.6 to 10.2% (Fig. 4). This suggests a close linkage between plant height and FLT. This is confirmed by the significant correlation between flag leaf thickness and plant height (*r* = 0.51) (Supplementary Figure S3a). However, when we grouped all the lines according to plant height, wide variations in FLT were shown in each group. For example, in the group with plant height between 85 and 95 (most likely to have the semi-dwarf gene), the FLT ranged from 220 to 320 (Supplementary Figure S3b). Therefore, this thick leaf QTL can be combined with the dwarf gene in breeding programs.

Based on correlation analysis, flag leaf thickness had significant positive correlations with grain weight, grain length, and seed area (Fig. 2). When using FLT as covariates, *Qpl-3H*, *Qgl-3H*, and *Qasa-3H* became insignificant. All results demonstrated the significant contribution of flag leaf thickness to yield-related traits in barley.

### Candidate genes for FLT

Leaf thickness scaled with cell sizes, cell wall thicknesses and the thicknesses of component mesophyll tissues, specifically with the size of palisade mesophyll cells [55, 56]. Increased palisade cell height leads to improved uptake of carbon dioxide (CO_2_) into mesophyll cells, and improved photosynthesis in thick leaves was witnessed [57]. Similarly, another study in the *Arabidopsis* Cvi ecotype found increased palisade mesophyll cell length contributed to increase of leaf thickness [58].

The QTL cluster regions of *Qflt-2H* and *Qflt-3H* not only affected the morphology of flag leaf but also had a positive effect on grain yield-related traits. From the annotation database (https://webblast.ipk-gatersleben.de/downloads/barley/), a gene that encodes LONGIFOLIA ½ protein (*HORVU.MOREX.r3.2HG0126960.1* (108, 708, 757˗108, 713, 879, Morex V3, 2021)) were found within the interval of *Qflt-2H* on chromosome 2H. Previously, a cell wall invertase gene *GIF1 (OsCIN2*) for rice has been reported to determine grain-filling, which also contributes to seed development. In *Arabidopsis*, *LONGIFOLIA1* and *LONGIFOLIA2* are two homologous genes, and regulate leaf morphology by positively promoting longitudinal polar cell elongation [59]. *HORVU.MOREX.r3.3HG0307040* (563, 634, 754˗563, 636, 989, Morex V3, 2021)) on chromosome 3H, encodes NAC domain proteins, which are associated with secondary wall thickening in *Arabidopsis* [60, 61]. Secondary walls also have specialized functions in determining pollen release from anther, and fiber elongation in seed trichomes [60, 62]. Therefore, *HORVU.MOREX.r3.2HG0126960* and *HORVU.MOREX.r3.3HG0307040* have potential impacts on FLT and seed development. Other genes, such as *HORVU.MOREX.r3.2HG0126390* (Gibberellin receptor GID1A), *HORVU.MOREX.r3.2HG0127670* (Vegetative cell wall protein gp1), and *HORVU.MOREX.r3.3HG0308590* (SAUR-like auxin-responsive protein family) within QTL intervals of *Qflt-2H* and *Qflt-3H* are listed in Supplementary Tables S2 and 3, respectively.

### Improving barley production by rewilding for lost superior traits

Wild barley has been reported to contain great genetic variation which provides valuable genetic resources for the improvement of cultivated barley [63–65]. During the process of domestication for selected agronomic traits many of the inherited traits, in particular, biotic and abiotic stress tolerance, may have been weakened or lost [52]. Barley is one of the major crops with many lost traits during domestication through artificial selection by breeders to meet human needs. Identification of these “missing” beneficial genes/alleles in wild barley species would provide promising genetic resources for barley breeding in the future. Recent research have paid more attention to discovering biotic and abiotic stress tolerance genes from wild relatives [66, 67] which have been successfully used in breeding programs [68]. Domestication also caused the loss of many morphological traits, such as leaf thickness, which are hard to select based on field performance. The relationship between leaf morphology and climate can evolve repeatedly in response to similar environments [15]. Results from our preliminary screening of 700 barley genotypes also supported the hypothesis with most of the 30 genotypes that have the thickest flag leaves (over 310 μm) being wild barley of landraces. In contrast, the FLT of commercial varieties was around 250 μm with several of them being around 200 μm (Supplementary Table S4). However, some wild barley type may also have much thinner flag leaves phenotypes. A recent report showed that the FLT of a wild barley AWCS276 was less than 150 μm [53].

During the process of selection under resource-poor environments, seedlings of species or ecotypes with greater leaf dry mass per unit area will be chosen for longer leaf longevity, rather than selection acting on growth rates themselves, such as internode length [69]. Even when grown under favourable conditions, the plants will keep low growth rates [70]. Species with higher leaf mass per leaf area (LMA) have thicker laminas, veins that protrude more, higher tissue density, or combinations of these [71, 72]. High-LMA species tend to achieve a longer average leaf lifespan in a variety of habitats [16]. Thicker, tougher leaves are the most common and general-purpose of plants defence [73]. However, long leaf lifespan may also be correlated with greater relative allocation to tannins, phenols, or other defensive compounds [74].

Although QTL have recently been reported, genes regulating FLT in barley have not been identified. The identification of the genes can greatly facilitate crop development [22, 53, 57]. In this study, we identified four QTL for flag leaf thickness with all the alleles for increasing leaf thickness being from the wild accession. Pan-genomics which contains multiple high-quality sequences to show genetic diversity is now widely used in crops, including rice [75, 76], wheat [77], barley [78], soybean [79], and maize [80]. However, barley pan-genomic studies so far have been limited mostly to cultivated accessions, with only a few wild species [78, 81]. Wild species as sources of novel genes now present great potential for crop improvement by reintroducing into modern cultivars [52]. The candidate genes for *Qflt-2H* and *Qflt-3H* were used to blast the recently released barley pan-genome [78] at IPK barley blast server (http://webblast.ipk-gatersleben.de/barley_ibsc/). One of them, *HORVU2Hr1G031980*, was only found in the Morex reference genome rather than other wild or landrace barley varieties. Therefore, more attention should be paid to the exploration and mining of favourable alleles in wild barley resources, including these alleles to improve leaf traits thus producing a better trade-off between sink and source.

In conclusion, for the first time a major QTL was identified for flag leaf thickness with the thick leaf allele from a wild barley accession, which has been lost during evolution. The introduction of the allele to cultivated barley could have a potential in significantly increasing grain yield by improving source for grain filling. It also presents a great opportunity for scientists to conduct their functional studies of leaves by incorporating genetic and molecular approaches.

## Materials and methods

### Plant material

A barley DH population consisting of 155 lines was derived from a cross between Australian malting barley cultivar Gairdner (*Hordeum vulgare* L.) (two-rowed and short stature and Syrian wild barley SYR01 (*Hordeum spontaneum*)(two-rowed and tall plant) obtained from China. The population was constructed by the Tasmanian Institute of Agriculture, University of Tasmania.

### Trials and trait measurements

Field trials were conducted at Mt Pleasant Laboratory in Tasmania, Australia (147°08’E, 41°280’S). In the field condition, fifteen seeds of each line were sown in a 0.6 m row with a row spacing of 0.25 m on 20 April 2019 and 25 April 2020. The field management followed the local farmers’ practices. In the glasshouse condition, five seeds of each line were sown in a 2-L pot filled with commercial potting mixture on 10 May 2020, at a spacing of 0.2 m between each pot. All the trials were carried out following a randomized complete block design with three replications. At the full-ripe stage, ten main panicles of each line were collected from field trials in both 2019 and 2020. Then the seeds were used for measuring thousand grain weight (TGW, g), grain length (GL, mm), grain width (GW, mm), grain thickness (GT, mm), and average seed area (ASA, mm^2^) by using the stand-alone digital image analyser with inbuilt software for image analysis in 2019 and 2020. Grains weight per panicle (GWP, g) was subsequently calculated. Flag leaf thickness (FLT, µm) was measured by a non-destructive leaf thickness instrument after anthesis in both glasshouse and the field in 2020, following the method previously described [22]. Taking the main vein as the vertical centre line of the blade, we measured the thickness of the left (x1) and right (x2) side of the middle part of the leaf blade and calculated the value of (x1 + x2)/2 as the thickness of the leaf blade. Flag leaf length (FLL, cm), width (FLW, cm) and panicle length (PL, cm) of the same plants were also measured. We followed the equation FLA = 0.69 × FLL × FLW to calculate the flag leaf area (FLA, cm^2^) [27]. Plant height (PH, cm) was measured from soil surface to the top of the spike excluding the awns in the field in 2019 and 2020.

### Statistical analysis

The narrow-sense heritability (*h*^*2*^) was estimated as *h*^*2*^ = V_g_/ (V_g_ +V_gei_/s + V_e_/sr), where V_g_, V_gei_, and V_e_ are the variance contributed by genotype, genotype-by-environment interaction, and residual error, respectively, while s is the number of environments and r is the number of replicates. The best linear unbiased predictions (BLUPs) for sink and source characteristics of each line across different environments were calculated using mixed linear models in the R package ‘lme4’ [82]. Then, the mean value of BLUPs of each line were used for statistical analysis and QTL mapping. The Pearson’s correlation coefficients were computed with the R package ‘Hmisc’.

### Genotyping and QTL mapping

Genomic DNA of each line was extracted and purified from approximately 100 mg leaf tissue via a modified cetyltrimethylammonium bromide (CTAB) method (Murray and Thompson 1980). Whole genome diversity array technology (DArT) and single nucleotide polymorphism (SNP) genotyping based on the 2017 Morex barley reference genome assembly were conducted by Diversity Arrays Technology (Canberra, Australia; https://www.diversityarrays.com). A total of around 22,000 DArT markers and 13,000 SNPs evenly distributed on the seven barley chromosomes were used to genotype the parents and 155 DH lines (Supplementary Figure S4). After filtering out low-quality markers (missing rate ≥ 10%) and those showing non-polymorphic in the two parents or progenies, a set of 8,334 DArT markers and 4,485 SNPs were generated. Then, the 12,819 markers were screened for similarities to remove redundant markers, and significantly distorted (*P* < 0.01) markers were also removed. Finally, 5052 markers were used for genetic map construction [64, 66] and QTL mapping. Genetic and physical positions of markers were aligned with the 2017 Morex barley reference genome assembly [83] as well as the most recent assembly [84] (Figure S5) using 2H as an example.

MapQTL 6.0 [85] was used for QTL analysis. The procedures of QTL detection have been well described before [86]. Briefly, the interval mapping (IM) function was applied for initial QTL scanning. A QTL was claimed to be significant at a LOD value higher than 3.0. Then the approximate multiple QTL model (MQM) was used for genetic background control. The percentage of total phenotypic variance explained by each QTL (*R*^*2*^) and the additive effect was obtained using the restricted MQM (rMQM) function. Interval of each QTL was calculated as the 95% confidence interval (1 LOD drop-off). MapChart 2.32 [87] was used for the graphical representation of linkage groups and QTL locations.

## Supplementary Information

**Additional file 1.**

**Additional file 2.**

**Additional file 3.**

## Data Availability

The datasets supporting the conclusions of this article are included within the article and its additional files. Original phenotypic data, genotypic data and molecular map file are included in additional files. Sub-section of all DH lines can be obtained from the corresponding author, Prof Meixue Zhou, TIA, University of Tasmania, under Material Transfer Agreement.
