# Supplementary material for: A new major QTL for flag leaf thickness in barley (Hordeum vulgare L.)
Source: BMC Plant Biol. 2022 Jun 24;22:305. doi: 10.1186/s12870-022-03694-7 (PMC9229122; doi:10.1186/s12870-022-03694-7)
Supplement: Supplementary file 2 — Additional file 2. [file 12870_2022_3694_MOESM2_ESM.pptx]

## Slide 1
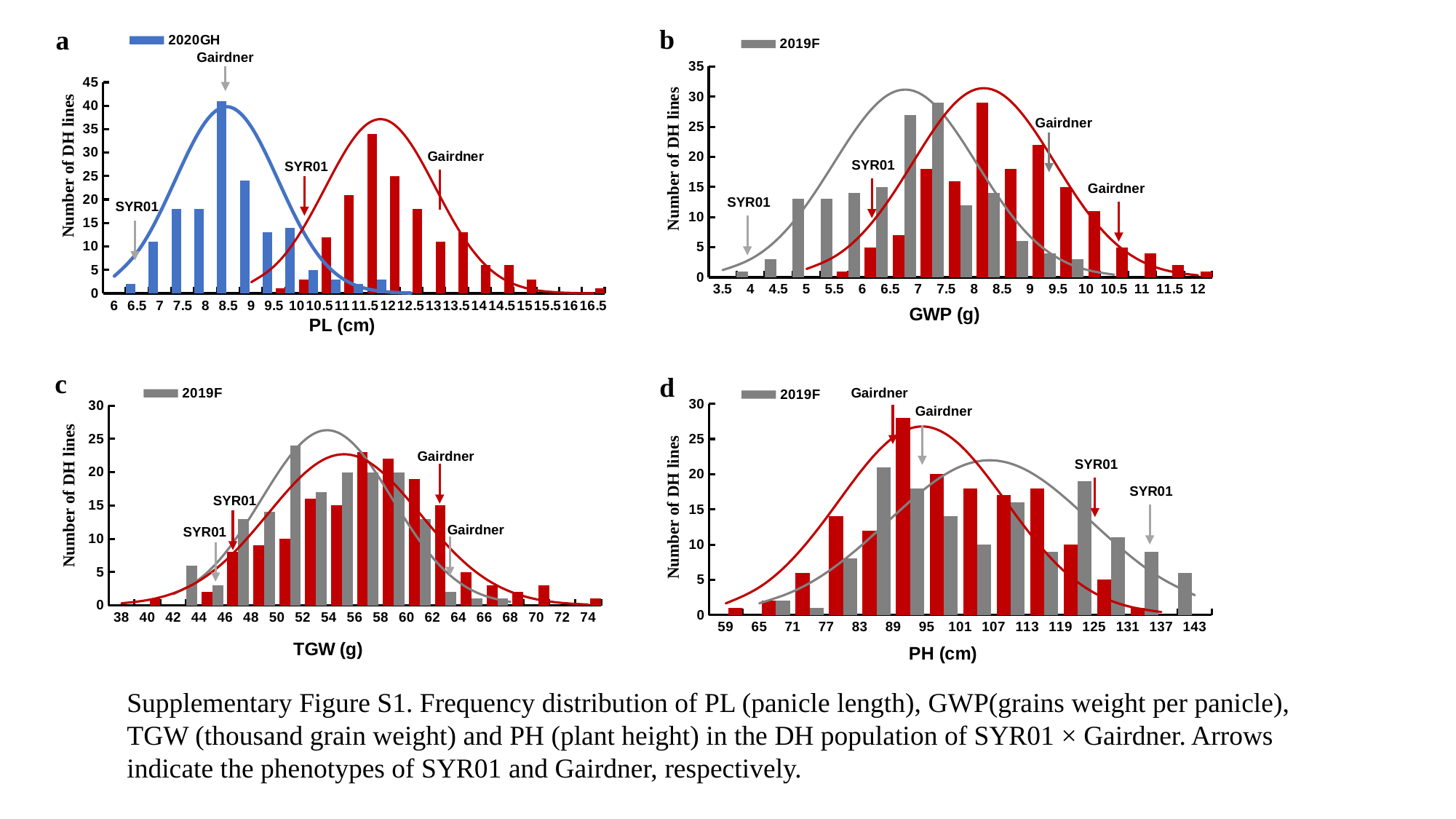

### Chart: GWP (g)
| Category | 2019F | 2020F | NORM.DIS_2019F | NORM.DIS_2020F |
|---|---|---|---|---|
| 3.5 | 0.0 | 0.0 | 0.012042536400404246 | None |
| 4 | 1.0 | 0.0 | 0.030235093005312515 | None |
| 4.5 | 3.0 | 0.0 | 0.06516010712370246 | None |
| 5 | 13.0 | 0.0 | 0.12053950674504078 | 0.013928197178090185 |
| 5.5 | 13.0 | 1.0 | 0.19140539287871522 | 0.03443986315070148 |
| 6 | 14.0 | 5.0 | 0.2608891810501735 | 0.07293136150133987 |
| 6.5 | 15.0 | 7.0 | 0.30523554666492686 | 0.1322676442366782 |
| 7 | 27.0 | 18.0 | 0.3065429181710629 | 0.20543730506936686 |
| 7.5 | 29.0 | 16.0 | 0.26425584721356765 | 0.2732696455287299 |
| 8 | 12.0 | 29.0 | 0.19553975365573284 | 0.3113077057971781 |
| 8.5 | 14.0 | 18.0 | 0.12420030469464445 | 0.30372094445795456 |
| 9 | 6.0 | 22.0 | 0.06771539223748846 | 0.2537733459736773 |
| 9.5 | 4.0 | 15.0 | 0.03169051341366434 | 0.1815949066527316 |
| 10 | 3.0 | 11.0 | 0.012730582965589004 | 0.11128784024126258 |
| 10.5 | 0.0 | 5.0 | 0.004389797102525738 | 0.05840878312359332 |
| 11 | 0.0 | 4.0 | None | 0.026253970677464284 |
| 11.5 | 0.0 | 2.0 | None | 0.01010644057476453 |
| 12 | 0.0 | 1.0 | None | 0.003331868486965794 |b
a
### Chart: PL (cm)
| Category | 2020GH | 2020F | NORM.DIS_GH | NORM.DIS_F |
|---|---|---|---|---|
| 6 | 0.0 | 0.0 | 0.03272342795626487 | None |
| 6.5 | 2.0 | 0.0 | 0.07800371547558543 | None |
| 7 | 11.0 | 0.0 | 0.15278549952014062 | None |
| 7.5 | 18.0 | 0.0 | 0.24590045866404106 | None |
| 8 | 18.0 | 0.0 | 0.3251972696911305 | None |
| 8.5 | 41.0 | 0.0 | 0.3533823007857198 | None |
| 9 | 24.0 | 0.0 | 0.3155390028852465 | 0.021215321260237865 |
| 9.5 | 13.0 | 1.0 | 0.23151101290831277 | 0.05133914183904844 |
| 10 | 14.0 | 3.0 | 0.1395727427323455 | 0.1046918942363027 |
| 10.5 | 5.0 | 12.0 | 0.06914167909473724 | 0.17990489124051184 |
| 11 | 3.0 | 21.0 | 0.028144243710000058 | 0.26051836558758895 |
| 11.5 | 2.0 | 34.0 | 0.009413466795011217 | 0.3179063327196758 |
| 12 | 3.0 | 25.0 | 0.002587140166835192 | 0.3269079437971076 |
| 12.5 | 0.0 | 18.0 | 0.0005842526270368447 | 0.28328085808062103 |
| 13 | 0.0 | 11.0 | None | 0.20685901150981756 |
| 13.5 | 0.0 | 13.0 | None | 0.12729083088164414 |
| 14 | 0.0 | 6.0 | None | 0.06600628011171622 |
| 14.5 | 0.0 | 6.0 | None | 0.028842895424783164 |
| 15 | 0.0 | 3.0 | None | 0.010620816687713383 |
| 15.5 | 0.0 | 0.0 | None | 0.003295660230936351 |
| 16 | 0.0 | 0.0 | None | 0.0008617720612283136 |
| 16.5 | 0.0 | 1.0 | None | 0.00018989251624731821 |Gairdner
Number of DH lines
Number of DH lines
Gairdner
SYR01
SYR01
Gairdner
SYR01
SYR01
### Chart: TGW (g)
| Category | 2019F | 2020F | NORM.DIS_2019F | NORM.DIS_2020F |
|---|---|---|---|---|
| 38 | 0.0 | 0.0 | None | 0.0009346827983344895 |
| 40 | 0.0 | 1.0 | None | 0.002395161010915448 |
| 42 | 0.0 | 0.0 | 0.005050927149250121 | 0.0054631603086412244 |
| 44 | 6.0 | 2.0 | 0.01180721664184022 | 0.01109154238121874 |
| 46 | 3.0 | 8.0 | 0.023603741906287563 | 0.02004373762470021 |
| 48 | 13.0 | 9.0 | 0.04035255895805076 | 0.03224068369386151 |
| 50 | 14.0 | 10.0 | 0.05899540710081566 | 0.04616029165425876 |
| 52 | 24.0 | 16.0 | 0.07376022317793121 | 0.05882631078701227 |
| 54 | 17.0 | 15.0 | 0.07886478795951214 | 0.06672882068486666 |
| 56 | 20.0 | 23.0 | 0.07211090665386473 | 0.06737426704395294 |
| 58 | 20.0 | 22.0 | 0.05638656908124262 | 0.06054989930370301 |
| 60 | 20.0 | 19.0 | 0.03770572384214027 | 0.048436367368737125 |
| 62 | 13.0 | 15.0 | 0.021562335184302818 | 0.034488036644031723 |
| 64 | 2.0 | 5.0 | 0.010544869304194631 | 0.02185768413538214 |
| 66 | 1.0 | 3.0 | 0.004410049945167311 | 0.01233044038137114 |
| 68 | 1.0 | 2.0 | 0.001577257905353518 | 0.006191441834046697 |
| 70 | 0.0 | 3.0 | None | 0.002767220551567271 |
| 72 | 0.0 | 0.0 | None | 0.0011008661529531125 |
| 74 | 0.0 | 1.0 | None | 0.00038981992313941833 |
### Chart: PH (cm)
| Category | 2019F | 2020F | NORM.DIS_2019F | NORM.DIS_2020F |
|---|---|---|---|---|
| 59 | 0.0 | 1.0 | None | 0.0016511086657459983 |
| 65 | 0.0 | 2.0 | 0.0016629533697837956 | 0.0039398755851942045 |
| 71 | 2.0 | 6.0 | 0.0033376243154344476 | 0.007993398484222106 |
| 77 | 1.0 | 14.0 | 0.00600504370124586 | 0.01378867467108688 |
| 83 | 8.0 | 12.0 | 0.009685370446501949 | 0.020223470754533176 |
| 89 | 21.0 | 28.0 | 0.014003533758256722 | 0.025219180078263983 |
| 95 | 18.0 | 20.0 | 0.018150157226257107 | 0.02673919567056498 |
| 101 | 14.0 | 18.0 | 0.021088441375223674 | 0.02410503802915421 |
| 107 | 10.0 | 17.0 | 0.021964934689248344 | 0.01847606186536861 |
| 113 | 16.0 | 18.0 | 0.020508631997353605 | 0.012040737811424255 |
| 119 | 9.0 | 10.0 | 0.017165830136013062 | 0.006671736671826194 |
| 125 | 19.0 | 5.0 | 0.012879952798583711 | 0.0031431622426314667 |
| 131 | 11.0 | 1.0 | 0.008663334328268345 | 0.0012590321092648014 |
| 137 | 9.0 | 0.0 | 0.005223687888375086 | 0.00042879421597262675 |
| 143 | 6.0 | 0.0 | 0.002823518959190808 | None |c
d
Gairdner
Gairdner
Number of DH lines
Number of DH lines
Gairdner
SYR01
SYR01
SYR01
Gairdner
SYR01
Supplementary Figure S1. Frequency distribution of PL (panicle length), GWP(grains weight per panicle), TGW (thousand grain weight) and PH (plant height) in the DH population of SYR01 × Gairdner. Arrows indicate the phenotypes of SYR01 and Gairdner, respectively.

## Slide 2
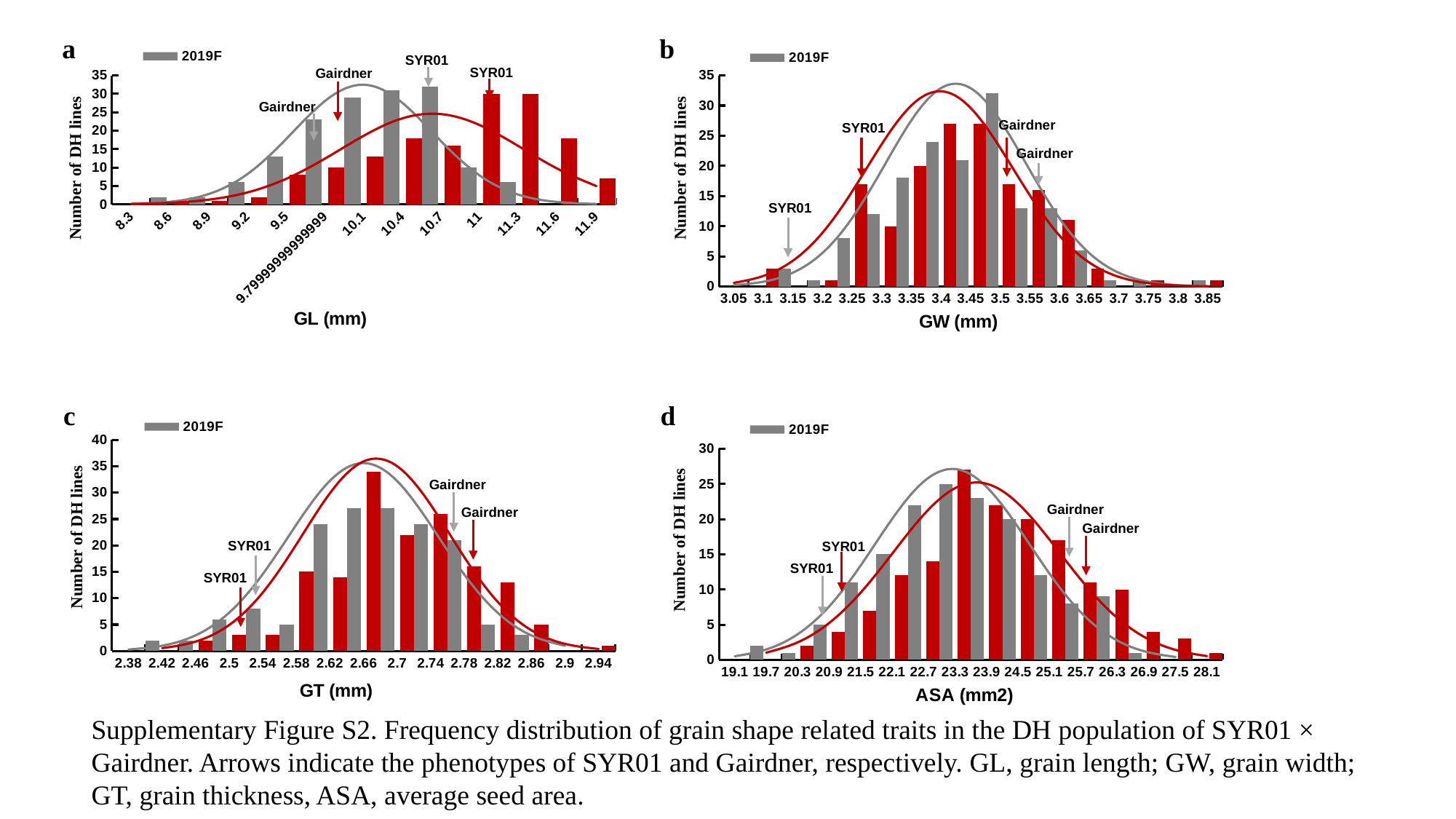

### Chart: GL (mm)
| Category | 2019F | 2020F | NORM.DIS_2019F | NORM.DIS_2020F |
|---|---|---|---|---|
| 8.3000000000000007 | 0.0 | 0.0 | 0.0029029440113589743 | 0.0025663304883059663 |
| 8.6 | 2.0 | 1.0 | 0.015929885383480193 | 0.009407382495738612 |
| 8.9 | 2.0 | 1.0 | 0.06402361039601613 | 0.028837440013160447 |
| 9.1999999999999993 | 6.0 | 2.0 | 0.1884608692253419 | 0.07392243917311893 |
| 9.5 | 13.0 | 8.0 | 0.4063083404734696 | 0.1584628840261008 |
| 9.7999999999999901 | 23.0 | 10.0 | 0.6415696389915732 | 0.28406027674074086 |
| 10.1 | 29.0 | 13.0 | 0.7419683646681486 | 0.42581910084584523 |
| 10.4 | 31.0 | 18.0 | 0.628464126097219 | 0.5337912544123695 |
| 10.7 | 32.0 | 16.0 | 0.389878345921955 | 0.5595635958129281 |
| 11 | 10.0 | 30.0 | 0.1771459615795305 | 0.4905227262660015 |
| 11.3 | 6.0 | 30.0 | 0.058950421690598706 | 0.3595841142186528 |
| 11.6 | 0.0 | 18.0 | 0.014367992571974878 | 0.22043144300507755 |
| 11.9 | 0.0 | 7.0 | 0.0025648312249254273 | 0.11299995563322716 |
### Chart: GW (mm)
| Category | 2019F | 2020F | NORM.DIS_2019F | NORM.DIS_2020F |
|---|---|---|---|---|
| 3.05 | 0.0 | 0.0 | 0.023070189435134628 | 0.06076426084997851 |
| 3.1 | 0.0 | 3.0 | 0.07983504561672596 | 0.17567291988661646 |
| 3.15 | 3.0 | 0.0 | 0.2313177463309795 | 0.4308093157769107 |
| 3.2 | 1.0 | 1.0 | 0.5611736682682952 | 0.8961671442197127 |
| 3.25 | 8.0 | 17.0 | 1.13987853168332 | 1.5813085342064226 |
| 3.3 | 12.0 | 10.0 | 1.9386205919997417 | 2.3668344268401555 |
| 3.35 | 18.0 | 20.0 | 2.760578022850641 | 3.0049884439124344 |
| 3.4 | 24.0 | 27.0 | 3.2913963898418985 | 3.236245058712201 |
| 3.45 | 21.0 | 27.0 | 3.285740857819327 | 2.9564031755245384 |
| 3.5 | 32.0 | 17.0 | 2.7463721440155533 | 2.290918215851493 |
| 3.55 | 13.0 | 16.0 | 1.9220222913389546 | 1.5058412039421947 |
| 3.6 | 13.0 | 11.0 | 1.126238611788397 | 0.8396000061007548 |
| 3.65 | 6.0 | 3.0 | 0.5525548081583394 | 0.3970903703270634 |
| 3.7 | 1.0 | 0.0 | 0.2269829675788727 | 0.15930519669318605 |
| 3.75 | 1.0 | 1.0 | 0.07806999194056535 | 0.054211846082989734 |
| 3.8 | 0.0 | 0.0 | 0.022482673671819477 | 0.015648838758185308 |
| 3.85 | 1.0 | 1.0 | 0.005421066524734209 | 0.00383171942288067 |a
b
SYR01
SYR01
Gairdner
Number of DH lines
Number of DH lines
Gairdner
Gairdner
SYR01
Gairdner
SYR01
### Chart: GT (mm)
| Category | 2019F | 2020F | NORM.DIS_2019F | NORM.DIS_2020F |
|---|---|---|---|---|
| 2.38 | 0.0 | 0.0 | 0.034270928469931926 | None |
| 2.42 | 2.0 | 0.0 | 0.12479125402464059 | 0.06556944855442086 |
| 2.46 | 2.0 | 2.0 | 0.3723871875390228 | 0.22346633850945435 |
| 2.5 | 6.0 | 3.0 | 0.910662837102889 | 0.6181398834528111 |
| 2.54 | 8.0 | 3.0 | 1.8250417359661357 | 1.3877955467281746 |
| 2.58 | 5.0 | 15.0 | 2.9973695420161373 | 2.528880362642184 |
| 2.62 | 24.0 | 14.0 | 4.0342246799257975 | 3.7402027859705145 |
| 2.66 | 27.0 | 34.0 | 4.449714804700011 | 4.489790591879297 |
| 2.7 | 27.0 | 22.0 | 4.0221343596172225 | 4.374426185577339 |
| 2.74 | 24.0 | 26.0 | 2.979430603326987 | 3.459235829046521 |
| 2.78 | 21.0 | 16.0 | 1.808682252901319 | 2.2202573643892816 |
| 2.82 | 5.0 | 13.0 | 0.8997950151759471 | 1.1566195768166379 |
| 2.86 | 3.0 | 5.0 | 0.36684042778021725 | 0.4890371246486952 |
| 2.9 | 0.0 | 0.0 | 0.1225640497046547 | 0.16782520664525488 |
| 2.94 | 0.0 | 1.0 | None | 0.046745157001217424 |
### Chart: ASA (mm2)
| Category | 2019F | 2020F | NORM.DIS_2019F | NORM.DIS_2020F |
|---|---|---|---|---|
| 19.100000000000001 | 0.0 | 0.0 | 0.005099031201331951 | None |
| 19.7 | 2.0 | 0.0 | 0.014823108710077502 | 0.010256793716212778 |
| 20.3 | 1.0 | 2.0 | 0.03648229521648776 | 0.024919581223108757 |
| 20.9 | 5.0 | 4.0 | 0.07601796660536259 | 0.05243502770680553 |
| 21.5 | 11.0 | 7.0 | 0.13410395281775428 | 0.09555510797300529 |
| 22.1 | 15.0 | 12.0 | 0.20028948292602694 | 0.1508127144804202 |
| 22.7 | 22.0 | 14.0 | 0.25325961772716066 | 0.2061454007409762 |
| 23.3 | 25.0 | 27.0 | 0.27112214022564274 | 0.24403998313930172 |
| 23.9 | 23.0 | 22.0 | 0.24572834562856657 | 0.2502072942247077 |
| 24.5 | 20.0 | 20.0 | 0.1885544406209964 | 0.22217264740025228 |
| 25.1 | 12.0 | 17.0 | 0.12249250283283365 | 0.17085703180779224 |
| 25.7 | 8.0 | 11.0 | 0.0673710766090039 | 0.1137959401590049 |
| 26.3 | 9.0 | 10.0 | 0.03137102451582981 | 0.06564056682865049 |
| 26.9 | 1.0 | 4.0 | 0.012367307300597099 | 0.032792121973862165 |
| 27.5 | 0.0 | 3.0 | 0.004127744788387186 | 0.014187907956638003 |
| 28.1 | 0.0 | 1.0 | None | 0.005316414668101252 |c
d
Number of DH lines
Number of DH lines
Gairdner
Gairdner
Gairdner
Gairdner
SYR01
SYR01
SYR01
SYR01
Supplementary Figure S2. Frequency distribution of grain shape related traits in the DH population of SYR01 × Gairdner. Arrows indicate the phenotypes of SYR01 and Gairdner, respectively. GL, grain length; GW, grain width; GT, grain thickness, ASA, average seed area.

## Slide 3
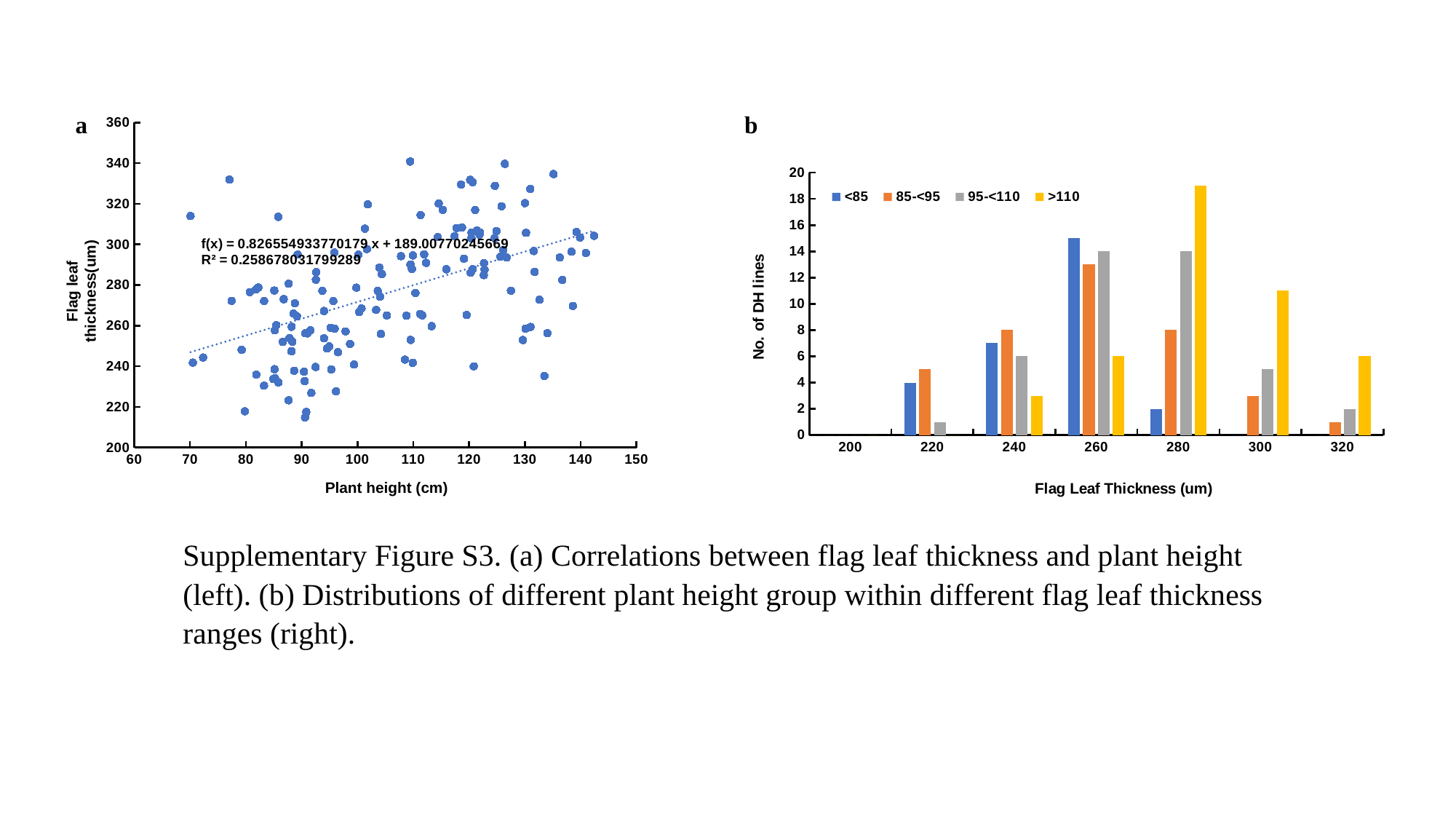

a
### Chart
| Category | <85 | 85-<95 | 95-<110 | >110 |
|---|---|---|---|---|
| 200 | 0.0 | 0.0 | 0.0 | 0.0 |
| 220 | 4.0 | 5.0 | 1.0 | 0.0 |
| 240 | 7.0 | 8.0 | 6.0 | 3.0 |
| 260 | 15.0 | 13.0 | 14.0 | 6.0 |
| 280 | 2.0 | 8.0 | 14.0 | 19.0 |
| 300 | 0.0 | 3.0 | 5.0 | 11.0 |
| 320 | 0.0 | 1.0 | 2.0 | 6.0 |b
### Chart
| Category | FieldFLTBLUEs |
|---|---|Flag leaf thickness(um)
Plant height (cm)
Supplementary Figure S3. (a) Correlations between flag leaf thickness and plant height (left). (b) Distributions of different plant height group within different flag leaf thickness ranges (right).

## Slide 4
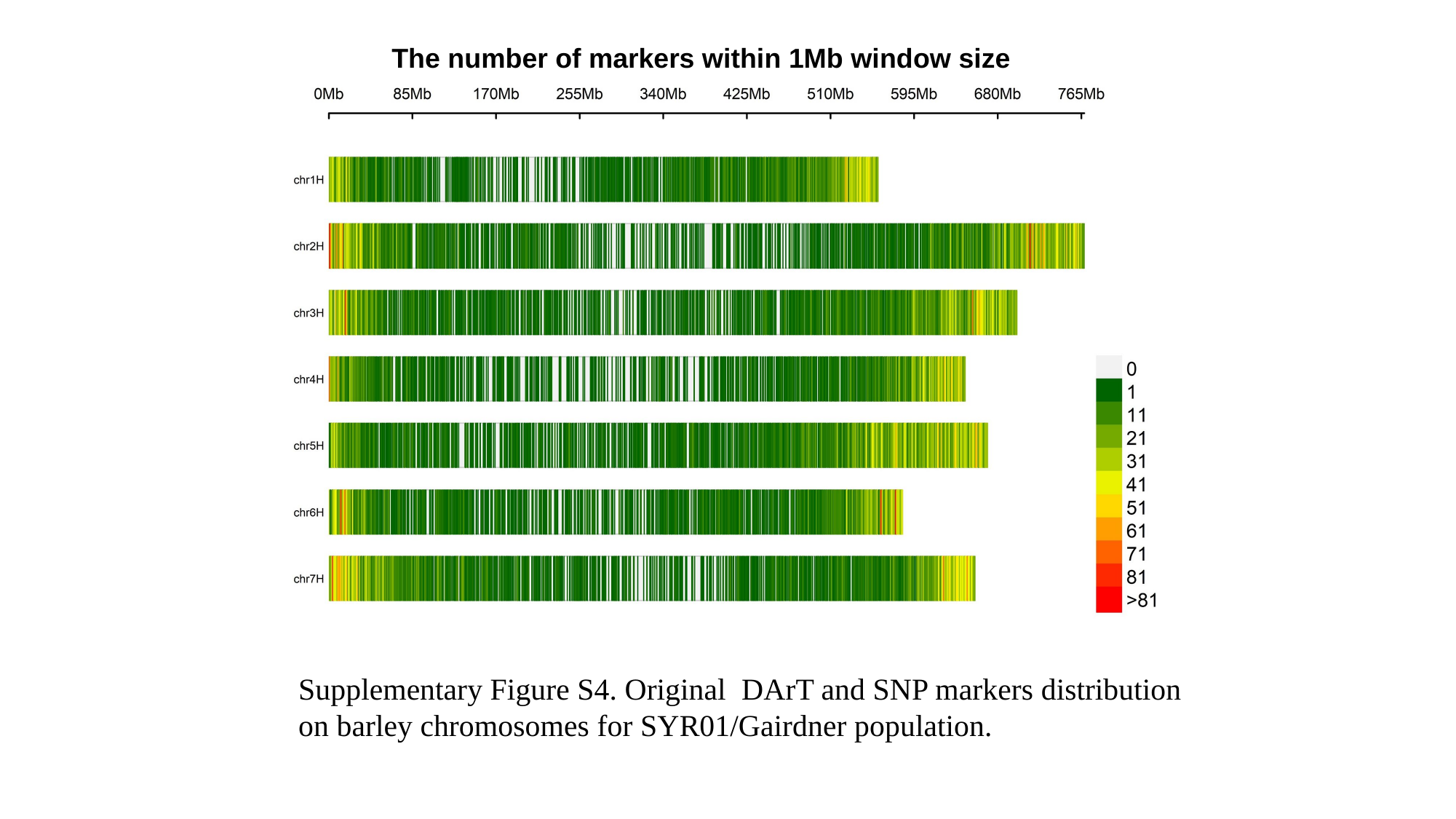

The number of markers within 1Mb window size
Supplementary Figure S4. Original DArT and SNP markers distribution on barley chromosomes for SYR01/Gairdner population.

## Slide 5
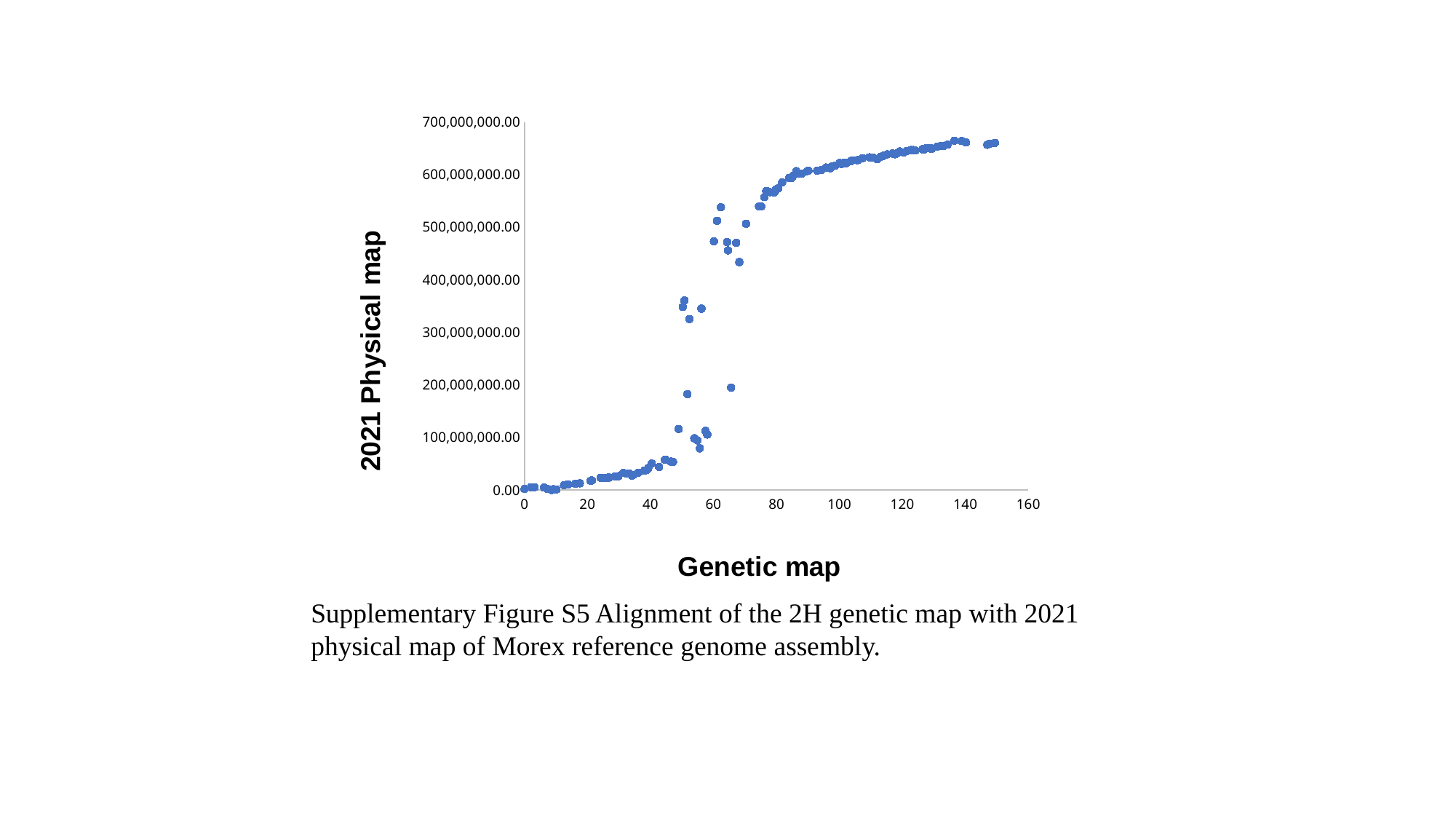

### Chart
| Category | |
|---|---|Supplementary Figure S5 Alignment of the 2H genetic map with 2021 physical map of Morex reference genome assembly.
