# Supplementary material for: A new major QTL for flag leaf thickness in barley (Hordeum vulgare L.)
Source: BMC Plant Biol. 2022 Jun 24;22:305. doi: 10.1186/s12870-022-03694-7 (PMC9229122; doi:10.1186/s12870-022-03694-7)
Supplement: Supplementary file 3 — Additional file 3. [file 12870_2022_3694_MOESM3_ESM.docx]

| Supplementary Table S1. Variance components and heritability estimates for flag leaf and yield related traits | | | | | | | | |  |  |  |  |  |  |  |
| --- | --- | --- | --- | --- | --- | --- | --- | --- | --- | --- | --- | --- | --- | --- | --- |
|  |  | |  | |  |  |  |  |  |  |  |  |  |  |  |
| Trait | V_g_ | | V_gei_ | | V_e_ | Replication | Environments | *h^2^* |  |  |  |  |  |  |  |
| FLT | 431.281 | | 236.311 | | 254.229 | 3 | 2 | 0.73 |  |  |  |  |  |  |  |
| FLL | 4.599 | | 0.809 | | 3.899 | 3 | 2 | 0.81 |  |  |  |  |  |  |  |
| FLW | 0.011 | | 0.003 | | 0.014 | 3 | 2 | 0.75 |  |  |  |  |  |  |  |
| FLA | 6.367 | | 2.446 | | 8.812 | 3 | 2 | 0.70 |  |  |  |  |  |  |  |
| PL | 0.999 | | 0.247 | | 0.331 | 3 | 2 | 0.85 |  |  |  |  |  |  |  |
| GWP | 0.705 | | 0.650 | | 0.765 | 3 | 2 | 0.61 |  |  |  |  |  |  |  |
| TGW | 13.679 | | 9.707 | | 17.897 | 3 | 2 | 0.64 |  |  |  |  |  |  |  |
| GL | 0.309 | | 0.036 | | 0.147 | 3 | 2 | 0.88 |  |  |  |  |  |  |  |
| GW | 0.008 | | 0.002 | | 0.013 | 3 | 2 | 0.73 |  |  |  |  |  |  |  |
| GT | 0.003 | | 0.001 | | 0.009 | 3 | 2 | 0.62 |  |  |  |  |  |  |  |
| ASA | 1.251 | | 0.617 | | 1.224 | 3 | 2 | 0.71 |  |  |  |  |  |  |  |
| V_g_: genotype variance; V_e_: residual error variance; V_gei_: genotype by environment interaction variance; *h^2^*: narrow-sense heritability.  Supplementary Table S2. Functional annotation of genes (Morex V3) within the QTL interval of *Qflt-2H.*   \| **Name of gene** \| **Physical position** \| **Functional annotation** \| \| --- \| --- \| --- \| \| HORVU.MOREX.r3.2HG0125550 \| 101511162-101514199 \| Elongation factor 1-beta \| \| HORVU.MOREX.r3.2HG0125560 \| 101514693-101515031 \| Calcium-dependent lipid-binding (CaLB domain) family protein \| \| HORVU.MOREX.r3.2HG0125570 \| 101516086-101516391 \| Protein FAR1-RELATED SEQUENCE 4 \| \| HORVU.MOREX.r3.2HG0125580 \| 101518174-101518734 \| Rap guanine nucleotide exchange factor 5 \| \| HORVU.MOREX.r3.2HG0125590 \| 101522263-101522628 \| RING/U-box superfamily protein \| \| HORVU.MOREX.r3.2HG0125600 \| 101528971-101530284 \| DNA-directed RNA polymerase subunit alpha \| \| HORVU.MOREX.r3.2HG0125610 \| 101532486-101533666 \| Urease accessory protein UreG 2 \| \| HORVU.MOREX.r3.2HG0125620 \| 101549860-101550678 \| Transposon protein \| \| HORVU.MOREX.r3.2HG0125630 \| 101556678-101564285 \| Inter-alpha-trypsin inhibitor heavy chain-like protein \| \| HORVU.MOREX.r3.2HG0125640 \| 101586038-101589508 \| Proteasome subunit alpha type \| \| HORVU.MOREX.r3.2HG0125650 \| 101589561-101591573 \| Adipocyte plasma membrane-associated protein \| \| HORVU.MOREX.r3.2HG0125660 \| 101984296-101985107 \| DUF1677 family protein (DUF1677) \| \| HORVU.MOREX.r3.2HG0125670 \| 102025901-102026209 \| Retrotransposon protein \| \| HORVU.MOREX.r3.2HG0125680 \| 102027981-102028361 \| ARM repeat superfamily protein \| \| HORVU.MOREX.r3.2HG0125690 \| 102030138-102030422 \| NADH-quinone oxidoreductase subunit B \| \| HORVU.MOREX.r3.2HG0125700 \| 102042544-102043329 \| Retrovirus-related Pol polyprotein from transposon TNT 1-94 \| \| HORVU.MOREX.r3.2HG0125710 \| 102053527-102053943 \| Retrovirus-related Pol polyprotein from transposon TNT 1-94 \| \| HORVU.MOREX.r3.2HG0125720 \| 102054079-102054738 \| Retrovirus-related Pol polyprotein from transposon TNT 1-94 \| \| HORVU.MOREX.r3.2HG0125730 \| 102060396-102062461 \| Zinc finger MYM-type-like protein \| \| HORVU.MOREX.r3.2HG0125740 \| 102097769-102098821 \| Retrovirus-related Pol polyprotein from transposon TNT 1-94 \| \| HORVU.MOREX.r3.2HG0125750 \| 102205169-102206444 \| Protein FAR1-RELATED SEQUENCE 5 \| \| HORVU.MOREX.r3.2HG0125760 \| 102206614-102208705 \| Protein FAR1-RELATED SEQUENCE 5 \| \| HORVU.MOREX.r3.2HG0125770 \| 102219111-102220457 \| Tyrosine recombinase XerC \| \| HORVU.MOREX.r3.2HG0125780 \| 102220487-102220966 \| Fructose-bisphosphate aldolase class 1 \| \| HORVU.MOREX.r3.2HG0125790 \| 102221678-102222671 \| F-box family protein \| \| HORVU.MOREX.r3.2HG0125800 \| 102222787-102224645 \| Replication protein A 32 kDa subunit \| \| HORVU.MOREX.r3.2HG0125810 \| 102227573-102231953 \| ATP synthase subunit b \| \| HORVU.MOREX.r3.2HG0125820 \| 102233084-102233705 \| Transducin/WD40 repeat-like superfamily protein \| \| HORVU.MOREX.r3.2HG0125830 \| 102237761-102238600 \| UPF0115 protein YfcN \| \| HORVU.MOREX.r3.2HG0125840 \| 102238899-102242536 \| Kinase family protein \| \| HORVU.MOREX.r3.2HG0125850 \| 102267804-102268172 \| 6,7-dimethyl-8-ribityllumazine synthase \| \| HORVU.MOREX.r3.2HG0125860 \| 102268360-102277345 \| Exportin-T \| \| HORVU.MOREX.r3.2HG0125870 \| 102489390-102490106 \| 50S ribosomal protein L15 \| \| HORVU.MOREX.r3.2HG0125880 \| 102494987-102498158 \| Rela/spot homolog 3 family protein \| \| HORVU.MOREX.r3.2HG0125890 \| 102501844-102504450 \| Transcription initiation factor TFIID subunit 9 \| \| HORVU.MOREX.r3.2HG0125900 \| 102777314-102778148 \| Transmembrane protein 53 \| \| HORVU.MOREX.r3.2HG0125910 \| 102844859-102845215 \| Retrotransposon protein, putative, Ty3-gypsy subclass \| \| HORVU.MOREX.r3.2HG0125920 \| 102845842-102848561 \| Alpha/beta-hydrolase superfamily protein \| \| HORVU.MOREX.r3.2HG0125930 \| 102850920-102852362 \| DNA-directed RNA polymerase III subunit \| \| HORVU.MOREX.r3.2HG0125940 \| 102961875-102964989 \| Methyltransferase-like protein \| \| HORVU.MOREX.r3.2HG0125950 \| 102966994-102967395 \| Polynucleotidyl transferase, ribonuclease H-like superfamily protein \| \| HORVU.MOREX.r3.2HG0125960 \| 102969185-102969448 \| Retrovirus-related Pol polyprotein from transposon TNT 1-94 \| \| HORVU.MOREX.r3.2HG0125970 \| 103024258-103024467 \| 3-octaprenyl-4-hydroxybenzoate carboxy-lyase \| \| HORVU.MOREX.r3.2HG0125980 \| 103037772-103038075 \| Serine/threonine-protein phosphatase 7 long form-like protein \| \| HORVU.MOREX.r3.2HG0125990 \| 103120817-103121093 \| C3H4 type zinc finger protein (DUF23) \| \| HORVU.MOREX.r3.2HG0126000 \| 103152479-103153488 \| Stress responsive alpha-beta barrel domain protein \| \| HORVU.MOREX.r3.2HG0126010 \| 103153816-103160280 \| RING/U-box superfamily protein \| \| HORVU.MOREX.r3.2HG0126020 \| 103165314-103176223 \| DNA mismatch repair protein mutS \| \| HORVU.MOREX.r3.2HG0126030 \| 103179445-103180269 \| LINE-1 reverse transcriptase-like protein \| \| HORVU.MOREX.r3.2HG0126040 \| 103181747-103182121 \| Polynucleotidyl transferase, ribonuclease H-like superfamily protein \| \| HORVU.MOREX.r3.2HG0126050 \| 103422130-103424472 \| 30S ribosomal protein S3 \| \| HORVU.MOREX.r3.2HG0126060 \| 103427491-103427826 \| cleavage and polyadenylation specificity factor 30 \| \| HORVU.MOREX.r3.2HG0126070 \| 103517122-103517490 \| Glutathione S-transferase T3 \| \| HORVU.MOREX.r3.2HG0126080 \| 103577596-103577925 \| Retrovirus-related Pol polyprotein from transposon TNT 1-94 \| \| HORVU.MOREX.r3.2HG0126090 \| 103621795-103627223 \| PHD finger alfin-like protein \| \| HORVU.MOREX.r3.2HG0126100 \| 103627682-103628074 \| Core-2/I-branching beta-1,6-N-acetylglucosaminyltransferase family protein \| \| HORVU.MOREX.r3.2HG0126110 \| 103715977-103718018 \| Retrotransposon protein, putative, unclassified, expressed \| \| HORVU.MOREX.r3.2HG0126120 \| 103719095-103719496 \| Retrotransposon protein, putative, unclassified \| \| HORVU.MOREX.r3.2HG0126130 \| 103720376-103720831 \| LINE-1 reverse transcriptase like \| \| HORVU.MOREX.r3.2HG0126140 \| 103721565-103722215 \| RNA-directed DNA polymerase (reverse transcriptase)-related family protein \| \| HORVU.MOREX.r3.2HG0126150 \| 103846296-103846598 \| Pentatricopeptide repeat (PPR) superfamily protein \| \| HORVU.MOREX.r3.2HG0126160 \| 103846938-103851067 \| alpha/beta-Hydrolases superfamily protein \| \| HORVU.MOREX.r3.2HG0126170 \| 103855362-103855839 \| ABRE binding factor 4 \| \| HORVU.MOREX.r3.2HG0126180 \| 103858579-103859082 \| Serine/threonine protein phosphatase 7 long form isogeny \| \| HORVU.MOREX.r3.2HG0126190 \| 103877502-103877822 \| Retrotransposon protein, putative, unclassified \| \| HORVU.MOREX.r3.2HG0126200 \| 103888724-103889089 \| GTPase Der \| \| HORVU.MOREX.r3.2HG0126210 \| 103890775-103892000 \| melanoma-associated antigen G1-like protein \| \| HORVU.MOREX.r3.2HG0126220 \| 104047963-104053988 \| Nuclear transcription factor Y subunit \| \| HORVU.MOREX.r3.2HG0126230 \| 104054097-104057367 \| 6,7-dimethyl-8-ribityllumazine synthase \| \| HORVU.MOREX.r3.2HG0126240 \| 104144786-104145166 \| B3 domain-containing protein \| \| HORVU.MOREX.r3.2HG0126250 \| 104159891-104160815 \| protein kinase family protein \| \| HORVU.MOREX.r3.2HG0126260 \| 104485725-104486722 \| Retrovirus-related Pol polyprotein from transposon TNT 1-94 \| \| HORVU.MOREX.r3.2HG0126270 \| 104488470-104490056 \| DUF2996 family protein \| \| HORVU.MOREX.r3.2HG0126280 \| 104493277-104496361 \| Acidic leucine-rich nuclear phosphoprotein 32-related protein \| \| HORVU.MOREX.r3.2HG0126290 \| 104621895-104622350 \| Crossover junction endodeoxyribonuclease RuvC \| \| HORVU.MOREX.r3.2HG0126300 \| 104779733-104780470 \| Embryogenesis transmembrane protein-like \| \| HORVU.MOREX.r3.2HG0126310 \| 104781920-104782258 \| Zinc finger (CCCH-type) family protein \| \| HORVU.MOREX.r3.2HG0126320 \| 104783268-104784151 \| MYB transcription factor \| \| HORVU.MOREX.r3.2HG0126330 \| 104907133-104907522 \| RNA-directed DNA polymerase (reverse transcriptase)-related family protein \| \| HORVU.MOREX.r3.2HG0126340 \| 104954592-104956969 \| sugar transporter, putative (DUF1195) \| \| HORVU.MOREX.r3.2HG0126350 \| 104977959-104979297 \| Pectinesterase \| \| HORVU.MOREX.r3.2HG0126360 \| 105153235-105160378 \| Agenet domain-containing protein \| \| HORVU.MOREX.r3.2HG0126370 \| 105268562-105269224 \| Acyl-CoA-binding domain-containing protein 4 \| \| HORVU.MOREX.r3.2HG0126380 \| 105431673-105433765 \| Heavy metal transport/detoxification superfamily protein \| \| HORVU.MOREX.r3.2HG0126390 \| 105551723-105553117 \| Gibberellin receptor GID1A \| \| HORVU.MOREX.r3.2HG0126400 \| 105571009-105571731 \| Retrovirus-related Pol polyprotein from transposon TNT 1-94 \| \| HORVU.MOREX.r3.2HG0126410 \| 105779890-105780982 \| Nuclear transcription factor Y subunit B \| \| HORVU.MOREX.r3.2HG0126420 \| 105863366-105864091 \| Recombination-associated protein RdgC \| \| HORVU.MOREX.r3.2HG0126430 \| 106071418-106072833 \| Serine/threonine protein phosphatase 7 long form isogeny \| \| HORVU.MOREX.r3.2HG0126440 \| 106074119-106082122 \| ATP-dependent RNA helicase-like protein \| \| HORVU.MOREX.r3.2HG0126450 \| 106084964-106086276 \| Sulfotransferase \| \| HORVU.MOREX.r3.2HG0126460 \| 106096771-106098006 \| Sulfotransferase \| \| HORVU.MOREX.r3.2HG0126470 \| 106098199-106098423 \| lysine ketoglutarate reductase trans-splicing-like protein (DUF707) \| \| HORVU.MOREX.r3.2HG0126480 \| 106216680-106217160 \| D-aminoacid aminotransferase-like PLP-dependent enzymes superfamily protein \| \| HORVU.MOREX.r3.2HG0126490 \| 106337224-106338062 \| Retrotransposon protein, putative, unclassified \| \| HORVU.MOREX.r3.2HG0126500 \| 106356592-106357011 \| Voltage-dependent calcium channel subunit alpha-2/delta-2 \| \| HORVU.MOREX.r3.2HG0126510 \| 106357328-106358243 \| EGG APPARATUS-1 protein \| \| HORVU.MOREX.r3.2HG0126520 \| 106590849-106591569 \| Formin-like protein \| \| HORVU.MOREX.r3.2HG0126530 \| 106880896-106881180 \| EGG APPARATUS-1 protein \| \| HORVU.MOREX.r3.2HG0126540 \| 106882708-106883112 \| EGG APPARATUS-1 protein \| \| HORVU.MOREX.r3.2HG0126550 \| 106887555-106892297 \| Alpha/beta hydrolase putative \| \| HORVU.MOREX.r3.2HG0126560 \| 107001986-107002300 \| Receptor-like protein kinase \| \| HORVU.MOREX.r3.2HG0126570 \| 107003272-107003766 \| Pentatricopeptide repeat-containing protein \| \| HORVU.MOREX.r3.2HG0126580 \| 107005560-107006113 \| Oligopeptidase A-like \| \| HORVU.MOREX.r3.2HG0126590 \| 107046381-107046716 \| B3 domain-containing transcription factorv rn1 \| \| HORVU.MOREX.r3.2HG0126600 \| 107072725-107073207 \| Retrovirus-related Pol polyprotein from transposon TNT 1-94 \| \| HORVU.MOREX.r3.2HG0126610 \| 107073211-107073834 \| Retrovirus-related Pol polyprotein from transposon TNT 1-94 \| \| HORVU.MOREX.r3.2HG0126620 \| 107074062-107074619 \| Retrovirus-related Pol polyprotein from transposon TNT 1-94 \| \| HORVU.MOREX.r3.2HG0126630 \| 107074860-107075399 \| Retrovirus-related Pol polyprotein from transposon TNT 1-94 \| \| HORVU.MOREX.r3.2HG0126640 \| 107087018-107089610 \| Transposon protein putative CACTA En/Spm sub-class \| \| HORVU.MOREX.r3.2HG0126650 \| 107093913-107094230 \| Sodium channel protein type 11 subunit alpha \| \| HORVU.MOREX.r3.2HG0126660 \| 107094605-107095057 \| Ribose-5-phosphate isomerase A \| \| HORVU.MOREX.r3.2HG0126670 \| 107095275-107095793 \| Aminotransferase-like plant mobile domain family protein \| \| HORVU.MOREX.r3.2HG0126680 \| 107096178-107097689 \| Myosin-10 \| \| HORVU.MOREX.r3.2HG0126690 \| 107120432-107120992 \| cGMP-specific 3' 5'-cyclic phosphodiesterase \| \| HORVU.MOREX.r3.2HG0126700 \| 107125178-107125639 \| Retrotransposon protein putative Ty1-copia subclass \| \| HORVU.MOREX.r3.2HG0126710 \| 107127704-107128249 \| Glucose-6-phosphate isomerase \| \| HORVU.MOREX.r3.2HG0126720 \| 107130425-107131051 \| Retrotransposon protein putative unclassified \| \| HORVU.MOREX.r3.2HG0126730 \| 107131117-107132980 \| Nuclear distribution protein nudE-like 1 \| \| HORVU.MOREX.r3.2HG0126740 \| 107133070-107133513 \| D-tagatose-1 6-bisphosphate aldolase subunit GatZ \| \| HORVU.MOREX.r3.2HG0126750 \| 107208454-107208990 \| Retrovirus-related Pol polyprotein from transposon TNT 1-94 \| \| HORVU.MOREX.r3.2HG0126760 \| 107294154-107302758 \| Histonedeacetylase \| \| HORVU.MOREX.r3.2HG0126760 \| 107294154-107302758 \| Histonedeacetylase \| \| HORVU.MOREX.r3.2HG0126770 \| 107499338-107499917 \| BTR1-like protein \| \| HORVU.MOREX.r3.2HG0126780 \| 107518137-107521500 \| SNF1-related protein kinase regulatory subunit gamma 1 \| \| HORVU.MOREX.r3.2HG0126780 \| 107518137-107521500 \| SNF1-related protein kinase regulatory subunit gamma 1 \| \| HORVU.MOREX.r3.2HG0126790 \| 107522052-107522897 \| Fasciclin-like arabinogalactan-protein-like \| \| HORVU.MOREX.r3.2HG0126800 \| 107670156-107671398 \| Fasciclin-like arabinogalactan-protein-like \| \| HORVU.MOREX.r3.2HG0126810 \| 107750453-107750818 \| Transposon protein putative Mutator sub-class \| \| HORVU.MOREX.r3.2HG0126820 \| 107835984-107837239 \| Fasciclin-like arabinogalactan-protein-like \| \| HORVU.MOREX.r3.2HG0126830 \| 107840346-107840795 \| Retrovirus-related Pol polyprotein from transposon TNT 1-94 \| \| HORVU.MOREX.r3.2HG0126840 \| 107843874-107845147 \| proteinaceous Rnase P 2 \| \| HORVU.MOREX.r3.2HG0126850 \| 107846567-107851072 \| RNA-binding protein \| \| HORVU.MOREX.r3.2HG0126860 \| 108051673-108055325 \| Receptor-like kinase \| \| HORVU.MOREX.r3.2HG0126870 \| 108056228-108057939 \| Glycerophosphodiester phosphodiesterase \| \| HORVU.MOREX.r3.2HG0126880 \| 108219396-108221707 \| Ninja-family protein \| \| HORVU.MOREX.r3.2HG0126890 \| 108324111-108324606 \| Lysine--tRNA ligase \| \| HORVU.MOREX.r3.2HG0126900 \| 108626181-108631864 \| GPI transamidase subunit PIG-U family protein \| \| HORVU.MOREX.r3.2HG0126910 \| 108697371-108701003 \| Nuclear transport factor 2 family protein with RNA binding domain \| \| HORVU.MOREX.r3.2HG0126920 \| 108703636-108704022 \| Epidermal growth factor receptor kinase substrate 8-like protein 2 \| \| HORVU.MOREX.r3.2HG0126930 \| 108705316-108705711 \| U3 small nucleolar RNA-associated protein 18-like protein \| \| HORVU.MOREX.r3.2HG0126940 \| 108705920-108706534 \| tRNA-2-methylthio-N(6)-dimethylallyladenosine synthase \| \| HORVU.MOREX.r3.2HG0126950 \| 108707685-108708050 \| DUF1336 family protein putative (DUF1336) \| \| HORVU.MOREX.r3.2HG0126960 \| 108708757-108713879 \| Longifolia protein \| \| HORVU.MOREX.r3.2HG0126970 \| 108733436-108733906 \| Transmembrane 9 super family member \| \| HORVU.MOREX.r3.2HG0126980 \| 108734231-108734800 \| Rho GDP-dissociation inhibitor \| \| HORVU.MOREX.r3.2HG0126990 \| 108740871-108741236 \| Retrovirus-related Pol polyprotein from transposon TNT 1-94 \| \| HORVU.MOREX.r3.2HG0127000 \| 108984081-108984422 \| Retrovirus-related Pol polyprotein from transposon TNT 1-94 \| \| HORVU.MOREX.r3.2HG0127010 \| 109036518-109037225 \| Octicosapeptide/Phox/Bem1p (PB1) domain-containing protein/ tetratricopeptide repeat (TPR)-containing protein \| \| HORVU.MOREX.r3.2HG0127020 \| 109404056-109408139 \| methyl esterase 11 \| \| HORVU.MOREX.r3.2HG0127030 \| 109419999-109420328 \| TTF-type zinc finger protein with HAT dimerization domain-containing protein \| \| HORVU.MOREX.r3.2HG0127040 \| 109571093-109572383 \| translocase inner membrane subunit 44-1 \| \| HORVU.MOREX.r3.2HG0127050 \| 109573406-109574199 \| spiral1 \| \| HORVU.MOREX.r3.2HG0127060 \| 109575272-109575808 \| Phthiocerol synthesis polyketide synthase type I PpsC \| \| HORVU.MOREX.r3.2HG0127070 \| 109773417-109773827 \| DNA polymerase epsilon catalytic subunit \| \| HORVU.MOREX.r3.2HG0127080 \| 109773885-109775023 \| Adenylate cyclase \| \| HORVU.MOREX.r3.2HG0127090 \| 109996736-109999114 \| Cytochrome P450 protein \| \| HORVU.MOREX.r3.2HG0127100 \| 110072346-110073743 \| Retrotransposon protein putative unclassified \| \| HORVU.MOREX.r3.2HG0127110 \| 110265580-110266035 \| bZIP domain class transcription factor (DUF630 and DUF632) \| \| HORVU.MOREX.r3.2HG0127120 \| 110267812-110268390 \| Retrotransposon protein putative Ty1-copia subclass \| \| HORVU.MOREX.r3.2HG0127130 \| 110323886-110327299 \| Peptide transporter \| \| HORVU.MOREX.r3.2HG0127140 \| 110330167-110333501 \| Pentatricopeptide repeat-containing protein putative \| \| HORVU.MOREX.r3.2HG0127140 \| 110330167-110333586 \| Pentatricopeptide repeat-containing protein putative \| \| HORVU.MOREX.r3.2HG0127150 \| 110566075-110572034 \| Glucosamine-6-phosphate deaminase \| \| HORVU.MOREX.r3.2HG0127150 \| 110566394-110572059 \| Glucosamine-6-phosphate deaminase \| \| HORVU.MOREX.r3.2HG0127160 \| 110615124-110616668 \| WD40 repeat-like protein \| \| HORVU.MOREX.r3.2HG0127170 \| 110670685-110671029 \| DnaJ protein ERDJ2A \| \| HORVU.MOREX.r3.2HG0127180 \| 110674409-110674930 \| Retrovirus-related Pol polyprotein from transposon TNT 1-94 \| \| HORVU.MOREX.r3.2HG0127190 \| 110676566-110677144 \| Retrovirus-related Pol polyprotein from transposon TNT 1-94 \| \| HORVU.MOREX.r3.2HG0127200 \| 110677223-110677630 \| Retrovirus-related Pol polyprotein from transposon TNT 1-94 \| \| HORVU.MOREX.r3.2HG0127210 \| 110678227-110678682 \| Retrovirus-related Pol polyprotein from transposon TNT 1-94 \| \| HORVU.MOREX.r3.2HG0127220 \| 110685619-110686112 \| Bifunctional inhibitor/lipid-transfer protein/seed storage 2S albumin super family protein \| \| HORVU.MOREX.r3.2HG0127230 \| 110689757-110691773 \| Nucleosome assembly protein 1-1 \| \| HORVU.MOREX.r3.2HG0127240 \| 110832885-110833124 \| Retrotransposon protein putative unclassified \| \| HORVU.MOREX.r3.2HG0127250 \| 110840648-110844319 \| Protein COBRA putative \| \| HORVU.MOREX.r3.2HG0127260 \| 110845456-110847441 \| COBRA-like protein \| \| HORVU.MOREX.r3.2HG0127270 \| 110850513-110850854 \| Mitochondrial import inner membrane translocase subunit Tim17 \| \| HORVU.MOREX.r3.2HG0127280 \| 110875985-110876389 \| Brefeldin A-inhibited guanine nucleotide-exchange protein 1 \| \| HORVU.MOREX.r3.2HG0127290 \| 110945069-110948961 \| Mitochondrial import inner membrane translocase subunit Tim17 \| \| HORVU.MOREX.r3.2HG0127300 \| 110947170-110947847 \| Mitochondrial import inner membrane translocase subunit Tim17 \| \| HORVU.MOREX.r3.2HG0127310 \| 111070542-111070733 \| Tripartite terminase subunit 3 \| \| HORVU.MOREX.r3.2HG0127320 \| 111073696-111074586 \| Tyrosine N-monooxygenase \| \| HORVU.MOREX.r3.2HG0127330 \| 111116995-111117792 \| B12D protein \| \| HORVU.MOREX.r3.2HG0127340 \| 111215772-111215996 \| Serine/threonine protein phosphatase 7 long form isogeny \| \| HORVU.MOREX.r3.2HG0127350 \| 111216621-111217193 \| Serine/threonine protein phosphatase 7 long form isogeny \| \| HORVU.MOREX.r3.2HG0127360 \| 111225070-111227297 \| PI-PLC X domain-containing protein \| \| HORVU.MOREX.r3.2HG0127370 \| 111270926-111274300 \| Alpha-1 4-glucan-protein synthase [UDP-forming] 1 \| \| HORVU.MOREX.r3.2HG0127380 \| 111479545-111480362 \| Ubiquitin carboxyl-terminal hydrolase 10 \| \| HORVU.MOREX.r3.2HG0127390 \| 111506894-111507446 \| Chitooligosaccharide deacetylase \| \| HORVU.MOREX.r3.2HG0127400 \| 111507467-111509122 \| Phosphoinositide phospholipase C \| \| HORVU.MOREX.r3.2HG0127410 \| 111586811-111611184 \| MADS box transcription factor \| \| HORVU.MOREX.r3.2HG0127410 \| 111586811-111611321 \| MADS-box transcription factor \| \| HORVU.MOREX.r3.2HG0127420 \| 111901483-111914280 \| Dihydroflavonol-4-reductase \| \| HORVU.MOREX.r3.2HG0127420 \| 111901483-111914280 \| Dihydroflavonol-4-reductase \| \| HORVU.MOREX.r3.2HG0127430 \| 111973326-111973868 \| Retrovirus-related Pol polyprotein from transposon TNT 1-94 \| \| HORVU.MOREX.r3.2HG0127440 \| 111974959-111975270 \| Retrovirus-related Pol polyprotein from transposon TNT 1-94 \| \| HORVU.MOREX.r3.2HG0127450 \| 111976685-111977026 \| Creatine kinase S-type mitochondrial \| \| HORVU.MOREX.r3.2HG0127460 \| 111987268-111988677 \| Retrovirus-related Pol polyprotein from transposon TNT 1-94 \| \| HORVU.MOREX.r3.2HG0127470 \| 111989234-111989593 \| Isoleucine--tRNA ligase \| \| HORVU.MOREX.r3.2HG0127480 \| 112017498-112020502 \| Anthocyanidin reductase \| \| HORVU.MOREX.r3.2HG0127490 \| 112088535-112088843 \| G-box-binding factor 1 \| \| HORVU.MOREX.r3.2HG0127500 \| 112298852-112302028 \| Dihydroflavonol-4-reductase \| \| HORVU.MOREX.r3.2HG0127510 \| 112356793-112357128 \| Myb/SANT-like DNA-binding domain protein \| \| HORVU.MOREX.r3.2HG0127520 \| 112358160-112358360 \| DWNN domain a CCHC-type zinc finger \| \| HORVU.MOREX.r3.2HG0127530 \| 112394040-112396892 \| Glucan endo-1 3-beta-glucosidase 3 \| \| HORVU.MOREX.r3.2HG0127540 \| 112488947-112494024 \| Ribosome biogenesis protein WDR12 \| \| HORVU.MOREX.r3.2HG0127550 \| 112494337-112495012 \| IgA FC receptor \| \| HORVU.MOREX.r3.2HG0127560 \| 112495873-112496073 \| Transposon protein putative Pong sub-class \| \| HORVU.MOREX.r3.2HG0127570 \| 112500713-112501247 \| IgA FC receptor \| \| HORVU.MOREX.r3.2HG0127580 \| 112502712-112504790 \| Delta-aminolevulinic acid dehydratase \| \| HORVU.MOREX.r3.2HG0127590 \| 112590229-112590892 \| mechanosensitive channel of small conductance-like 9 \| \| HORVU.MOREX.r3.2HG0127600 \| 112664289-112664720 \| IgA FC receptor \| \| HORVU.MOREX.r3.2HG0127610 \| 112665264-112665563 \| TTF-type zinc finger protein with HAT dimerization domain-containing protein \| \| HORVU.MOREX.r3.2HG0127620 \| 112665822-112667444 \| Zinc finger MYM-type-like protein \| \| HORVU.MOREX.r3.2HG0127630 \| 112671467-112671784 \| Hydroxyproline-rich glycoprotein family protein putative \| \| HORVU.MOREX.r3.2HG0127640 \| 112700860-112701423 \| IgA FC receptor \| \| HORVU.MOREX.r3.2HG0127650 \| 112754713-112755030 \| coiled-coil protein \| \| HORVU.MOREX.r3.2HG0127660 \| 112794281-112794770 \| Integrase-type DNA-binding superfamily protein \| \| HORVU.MOREX.r3.2HG0127670 \| 112795762-112796085 \| Vegetative cell wall protein gp1 \| \| HORVU.MOREX.r3.2HG0127680 \| 112804937-112805887 \| Retrovirus-related Pol poly protein from transposon TNT 1-94 \| \| HORVU.MOREX.r3.2HG0127690 \| 112819015-112819392 \| Seven transmembrane MLO family protein \| \| HORVU.MOREX.r3.2HG0127700 \| 112871489-112871983 \| myosin heavy chain embryonic smooth protein \| \| HORVU.MOREX.r3.2HG0127710 \| 112878819-112880111 \| peptide transporter 1 \| \| HORVU.MOREX.r3.2HG0127720 \| 112880557-112881152 \| C5a peptidase \| \| HORVU.MOREX.r3.2HG0127730 \| 112950465-112950791 \| Transposon protein putative CACTA En/Spm sub-class expressed \| \| HORVU.MOREX.r3.2HG0127740 \| 112957272-112957481 \| DNA helicase \| \| HORVU.MOREX.r3.2HG0127750 \| 113190727-113207984 \| Proteasome maturation factor UMP1 family protein \|   Supplementary Table S3. Functional annotation of genes (Morex V3) within the QTL interval of *Qflt-3H*. | | | | | | | | | | |  |  |  |  |  |
| **Name of gene** | | **Physical position** | | **Functional annotation** | | | | | |  |  |  |  |  |  |
| HORVU.MOREX.r3.3HG0306850 | | 563139825-563142630 | | UPF0301 protein | | | | | |  |  |  |  |  |  |
| HORVU.MOREX.r3.3HG0306860 | | 563153973-563154521 | | Ethylene-responsive transcription factor | | | | | |  |  |  |  |  |  |
| HORVU.MOREX.r3.3HG0306870 | | 563156299-563158423 | | nuclear pore anchor | | | | | |  |  |  |  |  |  |
| HORVU.MOREX.r3.3HG0306880 | | 563160105-563164708 | | 2-succinyl-6-hydroxy-2 4-cyclohexadiene-1-carboxylate synthase | | | | | |  |  |  |  |  |  |
| HORVU.MOREX.r3.3HG0306890 | | 563171189-563172076 | | DUF674 family protein | | | | | |  |  |  |  |  |  |
| HORVU.MOREX.r3.3HG0306900 | | 563230721-563231035 | | SAGA-associated factor 11 | | | | | |  |  |  |  |  |  |
| HORVU.MOREX.r3.3HG0306910 | | 563232101-563235465 | | TPX2 (Targeting protein for Xklp2) family protein putative | | | | | |  |  |  |  |  |  |
| HORVU.MOREX.r3.3HG0306920 | | 563311254-563313485 | | plasminogen activator inhibitor | | | | | |  |  |  |  |  |  |
| HORVU.MOREX.r3.3HG0306930 | | 563317111-563317422 | | Protoheme IX farnesyltransferase mitochondrial | | | | | |  |  |  |  |  |  |
| HORVU.MOREX.r3.3HG0306940 | | 563327335-563328042 | | RAC-like 1 | | | | | |  |  |  |  |  |  |
| HORVU.MOREX.r3.3HG0306950 | | 563328134-563328541 | | AP-5 complex subunit zeta-1 | | | | | |  |  |  |  |  |  |
| HORVU.MOREX.r3.3HG0306960 | | 563404329-563406537 | | Cytochrome c | | | | | |  |  |  |  |  |  |
| HORVU.MOREX.r3.3HG0306970 | | 563408066-563409319 | | B3 domain-containing protein | | | | | |  |  |  |  |  |  |
| HORVU.MOREX.r3.3HG0306980 | | 563454358-563454597 | | Transposon protein putative Pong sub-class | | | | | |  |  |  |  |  |  |
| HORVU.MOREX.r3.3HG0306990 | | 563456513-563459713 | | TVP38/TMEM64 family membrane protein | | | | | |  |  |  |  |  |  |
| HORVU.MOREX.r3.3HG0307000 | | 563459969-563461513 | | Pentatricopeptide repeat-containing protein | | | | | |  |  |  |  |  |  |
| HORVU.MOREX.r3.3HG0307010 | | 563462236-563466798 | | DNA replication complex GINS protein SLD5 | | | | | |  |  |  |  |  |  |
| HORVU.MOREX.r3.3HG0307020 | | 563548694-563550942 | | mesoderm induction early response protein | | | | | |  |  |  |  |  |  |
| HORVU.MOREX.r3.3HG0307030 | | 563570707-563580778 | | Zinc finger CCCH | | | | | |  |  |  |  |  |  |
| HORVU.MOREX.r3.3HG0307030 | | 563571006-563580650 | | Zinc finger CCCH | | | | | |  |  |  |  |  |  |
| HORVU.MOREX.r3.3HG0307040 | | 563634754-563636989 | | NAC domain-containing protein putative | | | | | |  |  |  |  |  |  |
| HORVU.MOREX.r3.3HG0307050 | | 563648080-563650454 | | Retrotransposon protein putative Ty3-gypsy subclass | | | | | |  |  |  |  |  |  |
| HORVU.MOREX.r3.3HG0307060 | | 563742032-563742607 | | XH/XS domain-containing protein | | | | | |  |  |  |  |  |  |
| HORVU.MOREX.r3.3HG0307070 | | 563743541-563744593 | | Transposon protein putative CACTA En/Spm sub-class | | | | | |  |  |  |  |  |  |
| HORVU.MOREX.r3.3HG0307080 | | 563757015-563757401 | | nine-cis-epoxycarotenoid dioxygenase 5 | | | | | |  |  |  |  |  |  |
| HORVU.MOREX.r3.3HG0307090 | | 563759878-563760312 | | beige/BEACH domain protein | | | | | |  |  |  |  |  |  |
| HORVU.MOREX.r3.3HG0307100 | | 563764010-563764444 | | Nuclease SbcCD subunit C | | | | | |  |  |  |  |  |  |
| HORVU.MOREX.r3.3HG0307110 | | 563777920-563778372 | | HAT transposon superfamily | | | | | |  |  |  |  |  |  |
| HORVU.MOREX.r3.3HG0307120 | | 563917226-563922459 | | S-adenosyl-L-methionine-dependentmethyltransferases superfamily protein | | | | | |  |  |  |  |  |  |
| HORVU.MOREX.r3.3HG0307130 | | 563923790-563926675 | | Gibberellin 20 oxidase 2 | | | | | |  |  |  |  |  |  |
| HORVU.MOREX.r3.3HG0307140 | | 564110797-564119506 | | Wound-responsive family protein | | | | | |  |  |  |  |  |  |
| HORVU.MOREX.r3.3HG0307150 | | 564121504-564123581 | | Glutathione S-transferase T3 | | | | | |  |  |  |  |  |  |
| HORVU.MOREX.r3.3HG0307160 | | 564126570-564128730 | | PISTILLATA-like MADS-box transcription factor | | | | | |  |  |  |  |  |  |
| HORVU.MOREX.r3.3HG0307170 | | 564129082-564131233 | | Receptor-like kinase | | | | | |  |  |  |  |  |  |
| HORVU.MOREX.r3.3HG0307180 | | 564165960-564166381 | | Kinesin-like protein | | | | | |  |  |  |  |  |  |
| HORVU.MOREX.r3.3HG0307190 | | 564166753-564171390 | | ATP-dependent DNA helicase pif1 | | | | | |  |  |  |  |  |  |
| HORVU.MOREX.r3.3HG0307200 | | 564260142-564260600 | | DNA topoisomerase | | | | | |  |  |  |  |  |  |
| HORVU.MOREX.r3.3HG0307210 | | 564263481-564263888 | | Glucose-6-phosphate isomerase | | | | | |  |  |  |  |  |  |
| HORVU.MOREX.r3.3HG0307220 | | 564270511-564271839 | | F-box family protein | | | | | |  |  |  |  |  |  |
| HORVU.MOREX.r3.3HG0307230 | | 564281258-564282271 | | Transposon protein putative Pong sub-class | | | | | |  |  |  |  |  |  |
| HORVU.MOREX.r3.3HG0307240 | | 564284581-564286688 | | Amino acid permease | | | | | |  |  |  |  |  |  |
| HORVU.MOREX.r3.3HG0307250 | | 564333893-564335978 | | Amino acid permease | | | | | |  |  |  |  |  |  |
| HORVU.MOREX.r3.3HG0307260 | | 564354964-564355245 | | Retrotransposon protein putative Ty3-gypsy subclass | | | | | |  |  |  |  |  |  |
| HORVU.MOREX.r3.3HG0307270 | | 564355442-564355747 | | Retrotransposon protein putative Ty3-gypsy subclass | | | | | |  |  |  |  |  |  |
| HORVU.MOREX.r3.3HG0307280 | | 564360158-564365804 | | Short-chain dehydrogenase/reductase family protein | | | | | |  |  |  |  |  |  |
| HORVU.MOREX.r3.3HG0307290 | | 564414451-564415607 | | NAD(P)H-quinone oxidoreductase subunit N | | | | | |  |  |  |  |  |  |
| HORVU.MOREX.r3.3HG0307300 | | 564415688-564416353 | | transmembrane protein putative (DUF679 domain membrane protein 1) | | | | | |  |  |  |  |  |  |
| HORVU.MOREX.r3.3HG0307310 | | 564416625-564420495 | | magnesiumtransporterputative (DUF803) | | | | | |  |  |  |  |  |  |
| HORVU.MOREX.r3.3HG0307320 | | 564427339-564427994 | | B3 domain-containing protein | | | | | |  |  |  |  |  |  |
| HORVU.MOREX.r3.3HG0307330 | | 564486944-564487881 | | ArfGap/RecO-like zinc finger | | | | | |  |  |  |  |  |  |
| HORVU.MOREX.r3.3HG0307340 | | 564607852-564611977 | | Receptor-like kinase | | | | | |  |  |  |  |  |  |
| HORVU.MOREX.r3.3HG0307350 | | 564651289-564651621 | | Glutathione S-transferase T3 | | | | | |  |  |  |  |  |  |
| HORVU.MOREX.r3.3HG0307360 | | 564659736-564663620 | | F-box family protein | | | | | |  |  |  |  |  |  |
| HORVU.MOREX.r3.3HG0307370 | | 564665210-564666326 | | B3 domain-containing protein | | | | | |  |  |  |  |  |  |
| HORVU.MOREX.r3.3HG0307380 | | 564666442-564666915 | | B3 domain-containing protein | | | | | |  |  |  |  |  |  |
| HORVU.MOREX.r3.3HG0307390 | | 564668561-564670021 | | 2-oxoglutarate (2OG) and Fe(II)-dependent oxygenase superfamily protein | | | | | |  |  |  |  |  |  |
| HORVU.MOREX.r3.3HG0307400 | | 564690110-564693849 | | GRAS family transcription factor | | | | | |  |  |  |  |  |  |
| HORVU.MOREX.r3.3HG0307410 | | 564693972-564696199 | | DNA ligase-like | | | | | |  |  |  |  |  |  |
| HORVU.MOREX.r3.3HG0307410 | | 564693972-564697773 | | DNA ligase-like | | | | | |  |  |  |  |  |  |
| HORVU.MOREX.r3.3HG0307420 | | 564700255-564703258 | | Bidirectional sugar transporter SWEET | | | | | |  |  |  |  |  |  |
| HORVU.MOREX.r3.3HG0307420 | | 564700255-564703258 | | Bidirectional sugar transporter SWEET | | | | | |  |  |  |  |  |  |
| HORVU.MOREX.r3.3HG0307420 | | 564700255-564703258 | | Bidirectional sugar transporter SWEET | | | | | |  |  |  |  |  |  |
| HORVU.MOREX.r3.3HG0307430 | | 564745661-564749641 | | Retrovirus-related Pol polyprotein from transposon TNT 1-94 | | | | | |  |  |  |  |  |  |
| HORVU.MOREX.r3.3HG0307440 | | 564777455-564781585 | | Membrane-associated 30 kDa protein chloroplasticon | | | | | |  |  |  |  |  |  |
| HORVU.MOREX.r3.3HG0307450 | | 564808977-564809213 | | Transposon protein putative Mutator sub-class | | | | | |  |  |  |  |  |  |
| HORVU.MOREX.r3.3HG0307460 | | 564811082-564812384 | | Aldose 1-epimerase | | | | | |  |  |  |  |  |  |
| HORVU.MOREX.r3.3HG0307470 | | 564819192-564830835 | | DNA/RNA helicase protein | | | | | |  |  |  |  |  |  |
| HORVU.MOREX.r3.3HG0307480 | | 564862557-564864146 | | Pentatricopeptide repeat-containing protein | | | | | |  |  |  |  |  |  |
| HORVU.MOREX.r3.3HG0307490 | | 564864759-564866328 | | Acyl-[acyl-carrier-protein] desaturase | | | | | |  |  |  |  |  |  |
| HORVU.MOREX.r3.3HG0307500 | | 564866426-564866776 | | ATP-dependent clp protease ATP-binding subunit clpx | | | | | |  |  |  |  |  |  |
| HORVU.MOREX.r3.3HG0307510 | | 564869466-564870293 | | BED zinc finger Hat family dimerization domain | | | | | |  |  |  |  |  |  |
| HORVU.MOREX.r3.3HG0307520 | | 564870326-564872030 | | bZIP transcription factor (DUF630 and DUF632) | | | | | |  |  |  |  |  |  |
| HORVU.MOREX.r3.3HG0307530 | | 564904854-564908790 | | Protein trichome birefringence | | | | | |  |  |  |  |  |  |
| HORVU.MOREX.r3.3HG0307540 | | 564938709-564941229 | | Pectinesterase | | | | | |  |  |  |  |  |  |
| HORVU.MOREX.r3.3HG0307550 | | 564951413-564951769 | | E3 ubiquitin-protein ligase MARCH11 | | | | | |  |  |  |  |  |  |
| HORVU.MOREX.r3.3HG0307560 | | 565021414-565027147 | | Hexosyltransferase | | | | | |  |  |  |  |  |  |
| HORVU.MOREX.r3.3HG0307570 | | 565035119-565035529 | | Retrotransposon protein putative Ty1-copia subclass | | | | | |  |  |  |  |  |  |
| HORVU.MOREX.r3.3HG0307580 | | 565042420-565042785 | | Cytokinin riboside 5'-monophosphate phosphoribohydrolase | | | | | |  |  |  |  |  |  |
| HORVU.MOREX.r3.3HG0307590 | | 565080183-565080551 | | Transposon protein Pong sub-class | | | | | |  |  |  |  |  |  |
| HORVU.MOREX.r3.3HG0307600 | | 565124030-565124509 | | Transposon protein putative Mariner sub-class | | | | | |  |  |  |  |  |  |
| HORVU.MOREX.r3.3HG0307610 | | 565222502-565223534 | | lysine-ketoglutarate reductase/saccharopine dehydrogenase bifunctional enzyme | | | | | |  |  |  |  |  |  |
| HORVU.MOREX.r3.3HG0307620 | | 565240212-565241792 | | DUF506 family protein | | | | | |  |  |  |  |  |  |
| HORVU.MOREX.r3.3HG0307630 | | 565330423-565332778 | | SET-domain protein lysine methyltransferase family protein | | | | | |  |  |  |  |  |  |
| HORVU.MOREX.r3.3HG0307640 | | 565346528-565347259 | | Retrotransposon protein putative unclassified | | | | | |  |  |  |  |  |  |
| HORVU.MOREX.r3.3HG0307650 | | 565518145-565519356 | | Transposon protein putative CACTA En/Spm sub-class expressed | | | | | |  |  |  |  |  |  |
| HORVU.MOREX.r3.3HG0307660 | | 565519658-565520721 | | Transposon protein putative CACTA En/Spm sub-class expressed | | | | | |  |  |  |  |  |  |
| HORVU.MOREX.r3.3HG0307670 | | 565526521-565528121 | | En/Spmtran sposon protein-like | | | | | |  |  |  |  |  |  |
| HORVU.MOREX.r3.3HG0307680 | | 565566690-565567166 | | Ribosomal protein L11 methyltransferase | | | | | |  |  |  |  |  |  |
| HORVU.MOREX.r3.3HG0307690 | | 565570158-565570541 | | RNA-directed DNA polymerase(reverse transcriptase)-related family protein | | | | | |  |  |  |  |  |  |
| HORVU.MOREX.r3.3HG0307700 | | 565585248-565585535 | | B3 domain-containing protein | | | | | |  |  |  |  |  |  |
| HORVU.MOREX.r3.3HG0307710 | | 565587999-565588361 | | Retrovirus-related Pol polyprotein from transposon TNT 1-94 | | | | | |  |  |  |  |  |  |
| HORVU.MOREX.r3.3HG0307720 | | 565591935-565592165 | | P-loop containing nucleoside triphosphate hydrolases superfamily protein | | | | | |  |  |  |  |  |  |
| HORVU.MOREX.r3.3HG0307730 | | 565601346-565603428 | | Mediator of RNA polymerase II transcription subunit 10 | | | | | |  |  |  |  |  |  |
| HORVU.MOREX.r3.3HG0307740 | | 565611717-565614590 | | RING/U-box superfamily protein | | | | | |  |  |  |  |  |  |
| HORVU.MOREX.r3.3HG0307750 | | 565672187-565673080 | | Retrovirus-related Pol polyprotein from transposon TNT 1-94 | | | | | |  |  |  |  |  |  |
| HORVU.MOREX.r3.3HG0307760 | | 565701348-565702620 | | 2-oxoglutarate and Fe(II)-dependent oxygenase superfamily protein putative | | | | | |  |  |  |  |  |  |
| HORVU.MOREX.r3.3HG0307770 | | 565704285-565704629 | | Malonyl CoA-acyl carrier protein transacylase | | | | | |  |  |  |  |  |  |
| HORVU.MOREX.r3.3HG0307780 | | 565705894-565706118 | | Histidine kinase | | | | | |  |  |  |  |  |  |
| HORVU.MOREX.r3.3HG0307790 | | 565725547-565727556 | | Retrovirus-related Pol polyprotein from transposon TNT 1-94 | | | | | |  |  |  |  |  |  |
| HORVU.MOREX.r3.3HG0307800 | | 565810983-565811918 | | Polynucleotidyl transferase ribonuclease H-like superfamily protein | | | | | |  |  |  |  |  |  |
| HORVU.MOREX.r3.3HG0307810 | | 565864356-565865291 | | Polynucleotidyl transferase ribonuclease H-like superfamily protein | | | | | |  |  |  |  |  |  |
| HORVU.MOREX.r3.3HG0307820 | | 565917697-565918632 | | Polynucleotidyl transferase ribonuclease H-like superfamily protein | | | | | |  |  |  |  |  |  |
| HORVU.MOREX.r3.3HG0307830 | | 565926587-565926892 | | 29 kDa protein | | | | | |  |  |  |  |  |  |
| HORVU.MOREX.r3.3HG0307840 | | 565935111-565935604 | | Myosin family XI heavy chain | | | | | |  |  |  |  |  |  |
| HORVU.MOREX.r3.3HG0307850 | | 565942527-565944065 | | Basic 7S globulin | | | | | |  |  |  |  |  |  |
| HORVU.MOREX.r3.3HG0307860 | | 565968493-565969962 | | Salicylate O-methyltransferase | | | | | |  |  |  |  |  |  |
| HORVU.MOREX.r3.3HG0307870 | | 565979988-565982812 | | F-box family protein | | | | | |  |  |  |  |  |  |
| HORVU.MOREX.r3.3HG0307880 | | 565987339-565988439 | | NAC domain protein | | | | | |  |  |  |  |  |  |
| HORVU.MOREX.r3.3HG0307890 | | 566052854-566053819 | | Peroxidase | | | | | |  |  |  |  |  |  |
| HORVU.MOREX.r3.3HG0307900 | | 566056159-566056743 | | DNA topoisomerase | | | | | |  |  |  |  |  |  |
| HORVU.MOREX.r3.3HG0307910 | | 566070822-566071787 | | Peroxidase | | | | | |  |  |  |  |  |  |
| HORVU.MOREX.r3.3HG0307920 | | 566074127-566074711 | | DNA topoisomerase | | | | | |  |  |  |  |  |  |
| HORVU.MOREX.r3.3HG0307930 | | 566110698-566111663 | | Peroxidase | | | | | |  |  |  |  |  |  |
| HORVU.MOREX.r3.3HG0307940 | | 566162004-566163995 | | Retrovirus-related Pol poly protein from transposon TNT 1-94 | | | | | |  |  |  |  |  |  |
| HORVU.MOREX.r3.3HG0307950 | | 566171679-566179492 | | GPI transamidase component PIG-S | | | | | |  |  |  |  |  |  |
| HORVU.MOREX.r3.3HG0307960 | | 566179960-566186637 | | Zinc finger protein | | | | | |  |  |  |  |  |  |
| HORVU.MOREX.r3.3HG0307970 | | 566191371-566197033 | | Sulfotransferase | | | | | |  |  |  |  |  |  |
| HORVU.MOREX.r3.3HG0307980 | | 566207177-566207873 | | Glutathione S-transferase T3 | | | | | |  |  |  |  |  |  |
| HORVU.MOREX.r3.3HG0307990 | | 566207980-566208255 | | ATP-dependent rRNA helicase SPB41 | | | | | |  |  |  |  |  |  |
| HORVU.MOREX.r3.3HG0308000 | | 566209389-566210848 | | Cysteine proteinase | | | | | |  |  |  |  |  |  |
| HORVU.MOREX.r3.3HG0308010 | | 566242156-566243626 | | Cysteine proteinase | | | | | |  |  |  |  |  |  |
| HORVU.MOREX.r3.3HG0308020 | | 566285575-566286696 | | Cysteine proteinase | | | | | |  |  |  |  |  |  |
| HORVU.MOREX.r3.3HG0308030 | | 566327690-566328811 | | Cysteine proteinase | | | | | |  |  |  |  |  |  |
| HORVU.MOREX.r3.3HG0308040 | | 566370998-566372119 | | Cysteine proteinase | | | | | |  |  |  |  |  |  |
| HORVU.MOREX.r3.3HG0308050 | | 566420191-566421312 | | Cysteine proteinase | | | | | |  |  |  |  |  |  |
| HORVU.MOREX.r3.3HG0308060 | | 566421414-566421806 | | Glutamine--fructose-6-phosphate aminotransferase [isomerizing] | | | | | |  |  |  |  |  |  |
| HORVU.MOREX.r3.3HG0308070 | | 566425791-566428868 | | TBC1 domain family member | | | | | |  |  |  |  |  |  |
| HORVU.MOREX.r3.3HG0308080 | | 566453099-566453747 | | RING/U-box superfamily protein | | | | | |  |  |  |  |  |  |
| HORVU.MOREX.r3.3HG0308090 | | 566471978-566472826 | | FAR1-related sequence 3 | | | | | |  |  |  |  |  |  |
| HORVU.MOREX.r3.3HG0308100 | | 566472926-566474202 | | FAR1-related sequence protein putative | | | | | |  |  |  |  |  |  |
| HORVU.MOREX.r3.3HG0308110 | | 566476202-566477965 | | Ubiquitin-like-specific protease ESD4 | | | | | |  |  |  |  |  |  |
| HORVU.MOREX.r3.3HG0308120 | | 566482134-566483495 | | Serine hydroxymethyltransferase | | | | | |  |  |  |  |  |  |
| HORVU.MOREX.r3.3HG0308130 | | 566500385-566500852 | | Connector enhancer of kinase suppressor of ras | | | | | |  |  |  |  |  |  |
| HORVU.MOREX.r3.3HG0308140 | | 566562677-566566153 | | Serine/arginine-rich splicing factor 1 | | | | | |  |  |  |  |  |  |
| HORVU.MOREX.r3.3HG0308150 | | 566630928-566631191 | | Serine/threonine protein phosphatase 7 long form isogeny | | | | | |  |  |  |  |  |  |
| HORVU.MOREX.r3.3HG0308160 | | 566631443-566631963 | | Transposon protein putative Mutator sub-class | | | | | |  |  |  |  |  |  |
| HORVU.MOREX.r3.3HG0308170 | | 566876786-566877373 | | zinc finger MYM-type-like protein | | | | | |  |  |  |  |  |  |
| HORVU.MOREX.r3.3HG0308180 | | 566878480-566881595 | | BTB/POZ and TAZ domain protein | | | | | |  |  |  |  |  |  |
| HORVU.MOREX.r3.3HG0308190 | | 566894918-566898501 | | BTB/POZ and TAZ domain protein | | | | | |  |  |  |  |  |  |
| HORVU.MOREX.r3.3HG0308200 | | 566901198-566901775 | | LINE-1 reverse transcriptase isogeny | | | | | |  |  |  |  |  |  |
| HORVU.MOREX.r3.3HG0308210 | | 566912544-566913071 | | Acyl carrier protein | | | | | |  |  |  |  |  |  |
| HORVU.MOREX.r3.3HG0308220 | | 567010376-567010813 | | Calcium-binding EF-hand family protein | | | | | |  |  |  |  |  |  |
| HORVU.MOREX.r3.3HG0308230 | | 567105285-567105848 | | Serine/threonine protein phosphatase 7 long form isogeny | | | | | |  |  |  |  |  |  |
| HORVU.MOREX.r3.3HG0308240 | | 567111491-567115182 | | Magnesium transporter MRS2-like protein | | | | | |  |  |  |  |  |  |
| HORVU.MOREX.r3.3HG0308250 | | 567126882-567133511 | | Telomere repeat-binding factor like-protein | | | | | |  |  |  |  |  |  |
| HORVU.MOREX.r3.3HG0308260 | | 567139606-567140149 | | Transcription factor gte4 | | | | | |  |  |  |  |  |  |
| HORVU.MOREX.r3.3HG0308270 | | 567159179-567161843 | | Proline transporter | | | | | |  |  |  |  |  |  |
| HORVU.MOREX.r3.3HG0308280 | | 567161619-567161918 | | Protein DETOXIFICATION | | | | | |  |  |  |  |  |  |
| HORVU.MOREX.r3.3HG0308290 | | 567162191-567165759 | | E3 ubiquitin-protein ligase RGLG2 | | | | | |  |  |  |  |  |  |
| HORVU.MOREX.r3.3HG0308300 | | 567185106-567185522 | | hAT family dimerization domain-containing protein | | | | | |  |  |  |  |  |  |
| HORVU.MOREX.r3.3HG0308310 | | 567186592-567187386 | | Zinc finger MYM-type protein 5 | | | | | |  |  |  |  |  |  |
| HORVU.MOREX.r3.3HG0308320 | | 567238668-567240416 | | Transposon protein putative CACTA En/Spm sub-class | | | | | |  |  |  |  |  |  |
| HORVU.MOREX.r3.3HG0308330 | | 567240771-567241391 | | Transposon protein putative CACTA En/Spm sub-class | | | | | |  |  |  |  |  |  |
| HORVU.MOREX.r3.3HG0308340 | | 567241530-567241979 | | Transposon protein putative CACTA En/Spm sub-class | | | | | |  |  |  |  |  |  |
| HORVU.MOREX.r3.3HG0308350 | | 567242542-567244155 | | Cysteine-rich receptor kinase | | | | | |  |  |  |  |  |  |
| HORVU.MOREX.r3.3HG0308360 | | 567269167-567273347 | | Cysteine-rich receptor kinase | | | | | |  |  |  |  |  |  |
| HORVU.MOREX.r3.3HG0308370 | | 567350759-567352232 | | Ubiquitin-like-specific protease ESD4 | | | | | |  |  |  |  |  |  |
| HORVU.MOREX.r3.3HG0308380 | | 567352736-567353698 | | Phosphatidylinositol 3 4 5-trisphosphate 3-phosphatase and dual-specific potein phosphatase PTEN | | | | | |  |  |  |  |  |  |
| HORVU.MOREX.r3.3HG0308390 | | 567360950-567361294 | | Retrovirus-related Pol polyprotein from transposon TNT 1-94 | | | | | |  |  |  |  |  |  |
| HORVU.MOREX.r3.3HG0308400 | | 567361613-567362758 | | Retrotransposon protein putative Ty1-copia subclass | | | | | |  |  |  |  |  |  |
| HORVU.MOREX.r3.3HG0308410 | | 567365789-567368951 | | cysteine-rich RECEPTOR-like kinase | | | | | |  |  |  |  |  |  |
| HORVU.MOREX.r3.3HG0308420 | | 567385258-567389755 | | Integrator complex subunit | | | | | |  |  |  |  |  |  |
| HORVU.MOREX.r3.3HG0308430 | | 567447017-567448501 | | Pentatricopeptide repeat-containing protein | | | | | |  |  |  |  |  |  |
| HORVU.MOREX.r3.3HG0308440 | | 567451678-567452178 | | Leucine-rich repeat receptor-like protein kinase family protein | | | | | |  |  |  |  |  |  |
| HORVU.MOREX.r3.3HG0308450 | | 567452650-567453312 | | Leucine-rich repeat receptor-like protein kinase family protein | | | | | |  |  |  |  |  |  |
| HORVU.MOREX.r3.3HG0308460 | | 567455512-567458064 | | Leucine-rich repeat receptor-like protein kinase family protein | | | | | |  |  |  |  |  |  |
| HORVU.MOREX.r3.3HG0308470 | | 567460040-567460381 | | Cysteine--tRNA ligase | | | | | |  |  |  |  |  |  |
| HORVU.MOREX.r3.3HG0308480 | | 567461188-567463278 | | Leucine-rich repeat receptor-like protein kinase family protein | | | | | |  |  |  |  |  |  |
| HORVU.MOREX.r3.3HG0308490 | | 567463399-567463722 | | mechanosensitive channel of small conductance-like 9 | | | | | |  |  |  |  |  |  |
| HORVU.MOREX.r3.3HG0308500 | | 567464707-567465153 | | tRNA1(Val) (adenine(37)-N6)-methyltransferase | | | | | |  |  |  |  |  |  |
| HORVU.MOREX.r3.3HG0308510 | | 567466029-567466566 | | low-molecular-weight cysteine-rich 55 | | | | | |  |  |  |  |  |  |
| HORVU.MOREX.r3.3HG0308520 | | 567497327-567498718 | | Late embryogenesis abundant (LEA) hydroxyproline-rich glycoprotein family member | | | | | |  |  |  |  |  |  |
| HORVU.MOREX.r3.3HG0308530 | | 567515953-567519432 | | Histone deacetylase putative | | | | | |  |  |  |  |  |  |
| HORVU.MOREX.r3.3HG0308540 | | 567520480-567523047 | | Histone deacetylase-like protein-like | | | | | |  |  |  |  |  |  |
| HORVU.MOREX.r3.3HG0308550 | | 567525432-567530022 | | Retrotransposon protein putative Ty3-gypsy subclass | | | | | |  |  |  |  |  |  |
| HORVU.MOREX.r3.3HG0308560 | | 567530885-567531103 | | Arginine--tRNA ligase | | | | | |  |  |  |  |  |  |
| HORVU.MOREX.r3.3HG0308570 | | 567531281-567533489 | | Fiber protein Fb34 | | | | | |  |  |  |  |  |  |
| HORVU.MOREX.r3.3HG0308580 | | 567645347-567654434 | | Dicer-like protein 3 | | | | | |  |  |  |  |  |  |
| HORVU.MOREX.r3.3HG0308590 | | 567767725-567768063 | | SAUR-like auxin-responsive protein family | | | | | |  |  |  |  |  |  |
| HORVU.MOREX.r3.3HG0308600 | | 567769265-567769741 | | Inorganic pyrophosphatase | | | | | |  |  |  |  |  |  |
| HORVU.MOREX.r3.3HG0308610 | | 567841969-567842454 | | RNA 3'-terminal phosphate cyclase | | | | | |  |  |  |  |  |  |
| HORVU.MOREX.r3.3HG0308620 | | 567848061-567852243 | | Protein prune | | | | | |  |  |  |  |  |  |
| HORVU.MOREX.r3.3HG0308630 | | 567853665-567855077 | | DUF868 family protein (DUF868) | | | | | |  |  |  |  |  |  |
| HORVU.MOREX.r3.3HG0308640 | | 567921039-567924237 | | lysine ketoglutarate reductase trans-splicing protein(DUF707) | | | | | |  |  |  |  |  |  |
| HORVU.MOREX.r3.3HG0308650 | | 567925117-567926340 | | Endosomal targeting BRO1-like domain-containing protein | | | | | |  |  |  |  |  |  |
| HORVU.MOREX.r3.3HG0308660 | | 567964990-567966195 | | F-box family protein | | | | | |  |  |  |  |  |  |
| HORVU.MOREX.r3.3HG0308670 | | 567968251-567968764 | | alpha/beta-Hydrolases superfamily protein | | | | | |  |  |  |  |  |  |
| HORVU.MOREX.r3.3HG0308680 | | 567971687-567972235 | | Coatomer beta' subunit | | | | | |  |  |  |  |  |  |
| HORVU.MOREX.r3.3HG0308690 | | 567973431-567975209 | | Retrotransposon protein putative Ty1-copia subclass | | | | | |  |  |  |  |  |  |
| HORVU.MOREX.r3.3HG0308700 | | 567976352-567977681 | | Retrotransposon protein putative Ty1-copia subclass | | | | | |  |  |  |  |  |  |
| HORVU.MOREX.r3.3HG0308710 | | 567980724-567981236 | | Beta-amylase | | | | | |  |  |  |  |  |  |
| HORVU.MOREX.r3.3HG0308720 | | 567994373-568004451 | | BSD domain (BTF2-like transcription factors Synapse-associated proteins) | | | | | |  |  |  |  |  |  |
| HORVU.MOREX.r3.3HG0308730 | | 568015734-568016105 | | FAR1 | | | | | |  |  |  |  |  |  |
| HORVU.MOREX.r3.3HG0308740 | | 568028234-568030923 | | RING/FYVE/PHD zinc finger protein putative | | | | | |  |  |  |  |  |  |
| HORVU.MOREX.r3.3HG0308750 | | 568038818-568039252 | | electron transfer flavoprotein beta | | | | | |  |  |  |  |  |  |
| HORVU.MOREX.r3.3HG0308760 | | 568039530-568039982 | | Maintenance of mitochondrial morphology protein 1 | | | | | |  |  |  |  |  |  |
| HORVU.MOREX.r3.3HG0308770 | | 568041458-568041994 | | Coiled-coil domain-containing protein 158 | | | | | |  |  |  |  |  |  |
| HORVU.MOREX.r3.3HG0308780 | | 568059406-568063887 | | ATP-dependent RNA helicase p62 | | | | | |  |  |  |  |  |  |
| HORVU.MOREX.r3.3HG0308780 | | 568059406-568063887 | | ATP-dependent RNA helicase p62 | | | | | |  |  |  |  |  |  |
| HORVU.MOREX.r3.3HG0308780 | | 568059406-568063897 | | ATP-dependent RNA helicase p62 | | | | | |  |  |  |  |  |  |
| HORVU.MOREX.r3.3HG0308790 | | 568291032-568291649 | | Ribonuclease HII 2 | | | | | |  |  |  |  |  |  |
| HORVU.MOREX.r3.3HG0308800 | | 568291808-568293220 | | F-box family protein | | | | | |  |  |  |  |  |  |
| HORVU.MOREX.r3.3HG0308810 | | 568297057-568299586 | | Collagen alpha-1(XXVII) chain A | | | | | |  |  |  |  |  |  |
| HORVU.MOREX.r3.3HG0308820 | | 568401123-568407674 | | Dolichyl-diphosphooligosaccharide--protein glycosyltransferase subunit 2 | | | | | |  |  |  |  |  |  |
| HORVU.MOREX.r3.3HG0308830 | | 568407963-568413012 | | ATP-binding ABC transporter | | | | | |  |  |  |  |  |  |
| HORVU.MOREX.r3.3HG0308840 | | 568458602-568462369 | | Aldehyde dehydrogenase putative | | | | | |  |  |  |  |  |  |
| HORVU.MOREX.r3.3HG0308840 | | 568458602-568462369 | | Aldehyde dehydrogenase putative | | | | | |  |  |  |  |  |  |
| HORVU.MOREX.r3.3HG0308840 | | 568458602-568462369 | | Aldehyde dehydrogenase putative | | | | | |  |  |  |  |  |  |
| HORVU.MOREX.r3.3HG0308850 | | 568474455-568474943 | | DNA replication helicase | | | | | |  |  |  |  |  |  |
| HORVU.MOREX.r3.3HG0308860 | | 568692389-568692736 | | Baculoviral IAP repeat-containing protein 5.2-B | | | | | |  |  |  |  |  |  |
| HORVU.MOREX.r3.3HG0308870 | | 568887143-568888070 | | Transposon protein putative CACTA En/Spm sub-class | | | | | |  |  |  |  |  |  |
| HORVU.MOREX.r3.3HG0308880 | | 569073396-569073854 | | Retrotransposon protein putative unclassified | | | | | |  |  |  |  |  |  |
| HORVU.MOREX.r3.3HG0308890 | | 569078898-569082740 | | Transcription factor viviparous 1 | | | | | |  |  |  |  |  |  |
| HORVU.MOREX.r3.3HG0308900 | | 569115430-569118100 | | F-box protein-like | | | | | |  |  |  |  |  |  |
| HORVU.MOREX.r3.3HG0308910 | | 569119970-569120722 | | AP2/B3-like transcriptional factor family protein | | | | | |  |  |  |  |  |  |
| HORVU.MOREX.r3.3HG0308920 | | 569152199-569156182 | | Retrovirus-related Pol polyprotein from transposon TNT 1-94 | | | | | |  |  |  |  |  |  |
| HORVU.MOREX.r3.3HG0308930 | | 569158428-569159302 | | Cortactin-binding protein 2 | | | | | |  |  |  |  |  |  |
| HORVU.MOREX.r3.3HG0308940 | | 569176478-569178532 | | Retrotransposon protein putative Ty1-copia subclass | | | | | |  |  |  |  |  |  |
| HORVU.MOREX.r3.3HG0308950 | | 569185885-569190048 | | F-box protein-like | | | | | |  |  |  |  |  |  |
| HORVU.MOREX.r3.3HG0308960 | | 569272286-569273849 | | Immediate early response 3-interacting 1 | | | | | |  |  |  |  |  |  |
| HORVU.MOREX.r3.3HG0308960 | | 569272317-569273849 | | Immediate early response 3-interacting 1 | | | | | |  |  |  |  |  |  |
| HORVU.MOREX.r3.3HG0308970 | | 569276274-569284114 | | Heavy meromyosin-like | | | | | |  |  |  |  |  |  |
| HORVU.MOREX.r3.3HG0308980 | | 569284632-569291952 | | S-adenosyl-L-methionine-dependent methyltransferase putative | | | | | |  |  |  |  |  |  |
| HORVU.MOREX.r3.3HG0308990 | | 569292179-569293192 | | MADS-box family protein | | | | | |  |  |  |  |  |  |
| HORVU.MOREX.r3.3HG0309000 | | 569299190-569304858 | | snRNA-activating protein complex subunit | | | | | |  |  |  |  |  |  |
| HORVU.MOREX.r3.3HG0309010 | | 569309951-569311619 | | Acyl-[acyl-carrier-protein] desaturase | | | | | |  |  |  |  |  |  |
| HORVU.MOREX.r3.3HG0309020 | | 569312982-569313188 | | Transposon protein putative Pong sub-class | | | | | |  |  |  |  |  |  |
| HORVU.MOREX.r3.3HG0309030 | | 569315460-569315693 | | Transposon protein putative Pong sub-class | | | | | |  |  |  |  |  |  |
| HORVU.MOREX.r3.3HG0309040 | | 569352426-569353897 | | Retrovirus-related Pol polyprotein from transposon TNT 1-94 | | | | | |  |  |  |  |  |  |
| HORVU.MOREX.r3.3HG0309050 | | 569365116-569366460 | | Werner Syndrome-like exonuclease | | | | | |  |  |  |  |  |  |
| HORVU.MOREX.r3.3HG0309060 | | 569389016-569390260 | | ATP-dependent DNA helicase PIF1 | | | | | |  |  |  |  |  |  |
| HORVU.MOREX.r3.3HG0309070 | | 569394383-569394700 | | Transcription factor jumonji (jmjC) domain-containing protein | | | | | |  |  |  |  |  |  |
| HORVU.MOREX.r3.3HG0309080 | | 569401789-569402682 | | MYB transcription factor | | | | | |  |  |  |  |  |  |
| HORVU.MOREX.r3.3HG0309090 | | 569416380-569419969 | | AT1G65230-like protein | | | | | |  |  |  |  |  |  |
| HORVU.MOREX.r3.3HG0309100 | | 569420734-569422218 | | Thioredoxin | | | | | |  |  |  |  |  |  |
| HORVU.MOREX.r3.3HG0309110 | | 569423025-569424584 | | Pentatricopeptide repeat-containing protein | | | | | |  |  |  |  |  |  |
| HORVU.MOREX.r3.3HG0309120 | | 569425287-569425616 | | Protein kinase superfamily protein | | | | | |  |  |  |  |  |  |
| HORVU.MOREX.r3.3HG0309130 | | 569431891-569433042 | | transmembrane protein putative (Protein of unknown function DUF538) | | | | | |  |  |  |  |  |  |
| HORVU.MOREX.r3.3HG0309140 | | 569547718-569551630 | | NRT1/PTR family protein 2.2 | | | | | |  |  |  |  |  |  |
| HORVU.MOREX.r3.3HG0309150 | | 569548958-569557141 | | RNA binding protein | | | | | |  |  |  |  |  |  |
| HORVU.MOREX.r3.3HG0309160 | | 569587010-569587303 | | Retrovirus-related Pol polyprotein from transposon TNT 1-94 | | | | | |  |  |  |  |  |  |
| HORVU.MOREX.r3.3HG0309170 | | 569595017-569599124 | | Glyceraldehyde-3-phosphate dehydrogenase | | | | | |  |  |  |  |  |  |
| HORVU.MOREX.r3.3HG0309180 | | 569612995-569613285 | | Gap junction alpha-1 protein | | | | | |  |  |  |  |  |  |
| HORVU.MOREX.r3.3HG0309190 | | 569633166-569635397 | | Pentatricopeptide repeat-containing protein | | | | | |  |  |  |  |  |  |

Supplementary Table S4. FLT distribution of 30 wild/landrace barley varieties and 16 commercial barley varieties

| ID | Type | FLT (µm) |
| --- | --- | --- |
| Xiaojiang | Landrace | 318 |
| HB10315 | Landrace | 318 |
| Cape | Landrace | 318 |
| SR440 | Landrace | 318 |
| BM9831-42 | Landrace | 320 |
| XZ115 | Wild barley | 321 |
| SHEBA | Landrace | 322 |
| CI5791 | Landrace | 323 |
| 48 UM | Landrace | 325 |
| IG 120489 | Landrace | 326 |
| ETHIOPIA 602 | Landrace | 326 |
| W2 | Landrace | 326 |
| Gobermadora | Landrace | 327 |
| Etu | Landrace | 328 |
| Russian 81 | Landrace | 330 |
| Dabat 5 | Landrace | 331 |
| BENTON | Landrace | 332 |
| B523 | Landrace | 332 |
| Kamadas 1 | Landrace | 335 |
| 137 UM | Landrace | 335 |
| WIEBE GA 148-3 | Landrace | 336 |
| X118 | Wild barley | 341 |
| 21 UM | Landrace | 342 |
| SB070206 | Landrace | 342 |
| KAJSA | Landrace | 345 |
| SR443 | Landrace | 349 |
| H94034003 | Landrace | 353 |
| CI5831 | Landrace | 364 |
| H94084001 | Landrace | 386 |
| SR439 | Landrace | 416 |
| Baudin | Commercial variety | 227 |
| Buloke | Commercial variety | 203 |
| Flagship | Commercial variety | 208 |
| Fleet | Commercial variety | 293 |
| Franklin | Commercial variety | 264 |
| Gairdner | Commercial variety | 263 |
| Hindmarsh | Commercial variety | 210 |
| Maritime | Commercial variety | 231 |
| Morex | Commercial variety | 234 |
| Planet | Commercial variety | 221 |
| Skiff | Commercial variety | 232 |
| Urambie | Commercial variety | 211 |
| Yangpi 1 | Commercial variety | 192 |
| YERONG | Commercial variety | 245 |
| YSM1 | Commercial variety | 230 |
| YSM3 | Commercial variety | 258 |
